# Supplementary material for: Mithramycin alters EWS::FLI1 DNA binding and RNA polymerase II processivity to inhibit nascent transcription
Source: Nat Commun. 2026 Feb 16;17:2844. doi: 10.1038/s41467-026-69488-9 (PMC13021929; doi:10.1038/s41467-026-69488-9)
Supplement: Supplementary file 1 — Supplementary Information [file 41467_2026_69488_MOESM1_ESM.pdf]

Supplementary Figures

S1

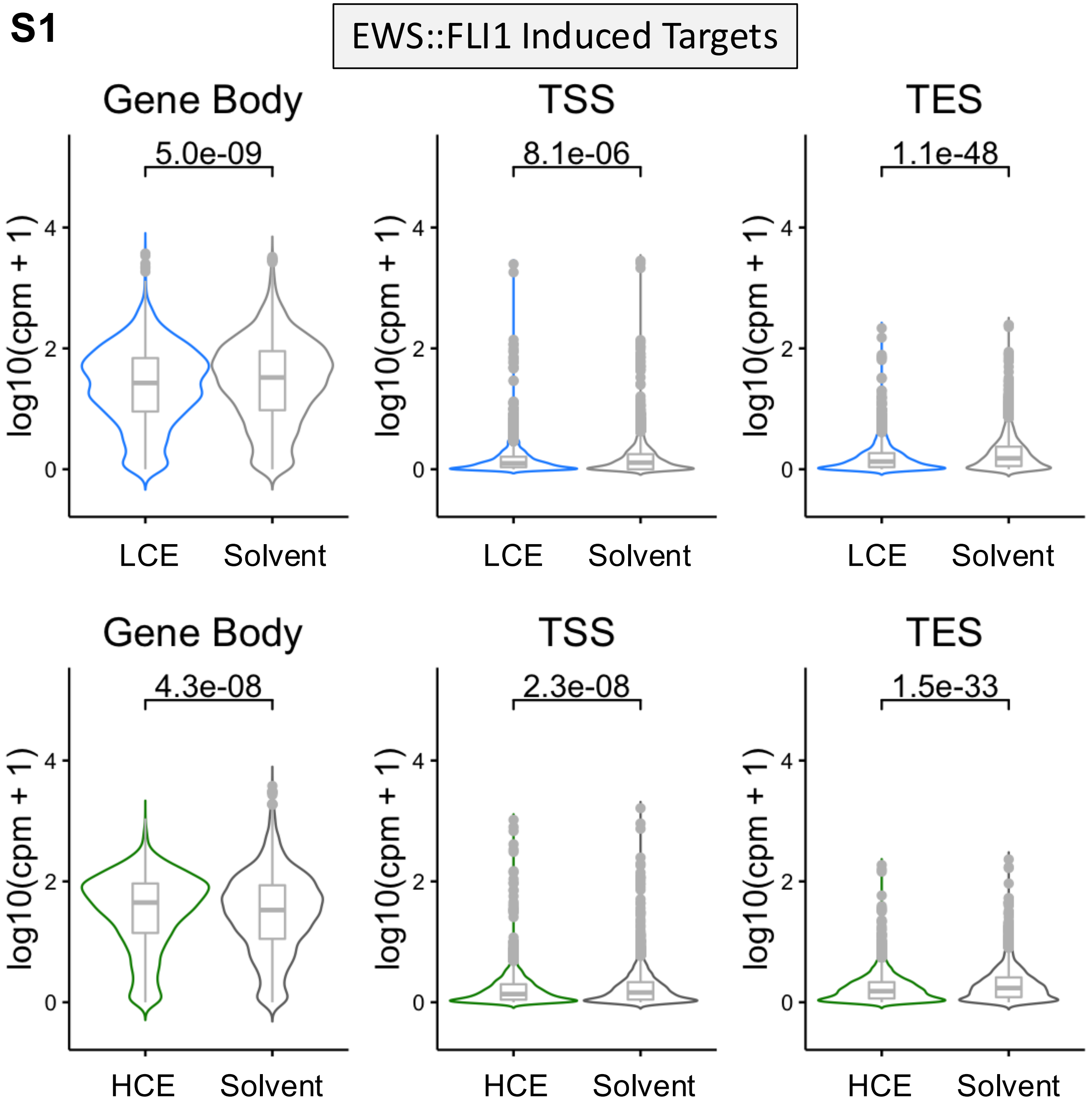

Figure S1: Quantitation of the effect of mithramycin on transcription elongation for EWS::FLI1 induced target genes. Violin plot of normalized counts Log_{10}(cpm+1) of EWS::FLI1 induced downstream targets at the Transcription Start Site (TSS), Gene Body, or Transcription End Site (TES) following LCE (20 nmol/L for 72 hours, top) or HCE (100 nmol/L for 18 hours, bottom) to MMA. Data shows statistically significant repression of EWS::FLI1 induced genes with LCE at the TSS, Gene Body and TES. HCE causes increased transcription initiation evident in the Gene Body but overall suppression at the TES due to impairment of RNAPII processivity. Data represents a two-sided Wilcoxon rank-sum test comparing biological replicates of solvent control (n = 3) to LCE (n = 2) or different solvent controls (n = 3) to HCE (n =3). Center lines show medians, box limits indicate 25th to 75th percentile (IQR), and whiskers extend 1.5 times the IQR (see source data file for complete numbers and statistics).

S2

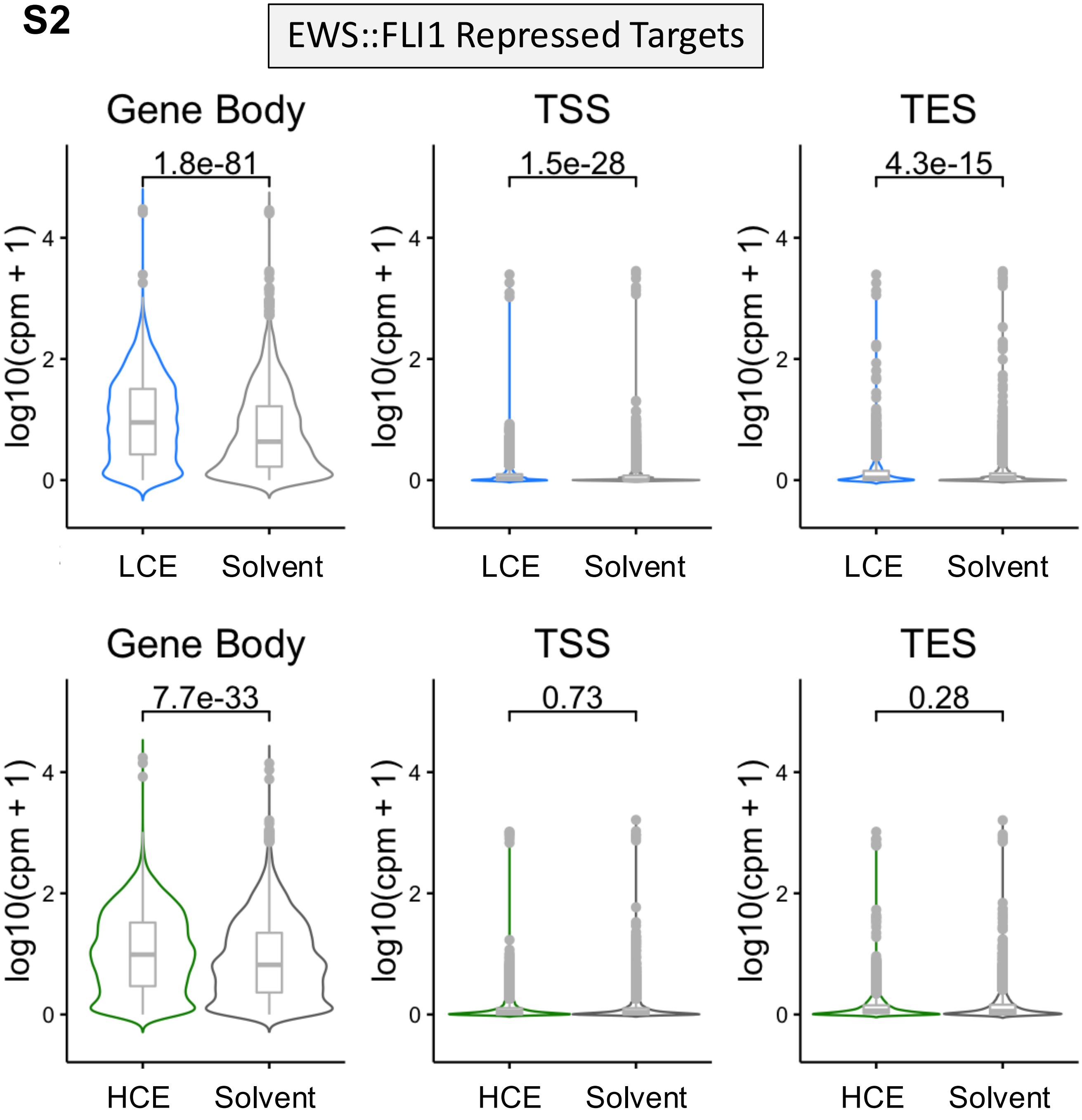

Figure S2: Quantitation of the effect of mithramycin on transcription elongation for EWS::FLI1 repressed target genes. Violin plot of normalized counts Log_{10}(cpm+1) of EWS::FLI1 repressed downstream targets at the Transcription Start Site (TSS), Gene Body, or Transcription End Site (TES) following LCE or HCE to MMA. There is statistically significant induction of EWS::FLI1 repressed genes with LCE at the TSS, Gene Body and TES. HCE causes increased transcription initiation evident in the Gene Body but is unable to fully induce these repressed targets to complete productive transcription at the TES due to impaired RNAPII processivity. Data represents a two-sided Wilcoxon rank-sum test comparing biological replicates of solvent control (n = 3) to LCE (n = 2) or different solvent controls (n = 3) to HCE (n =3). Center lines show medians, box limits indicate 25th to 75th percentile (IQR), and whiskers extend 1.5 times the IQR (see source data file for complete numbers and statistics).

S3

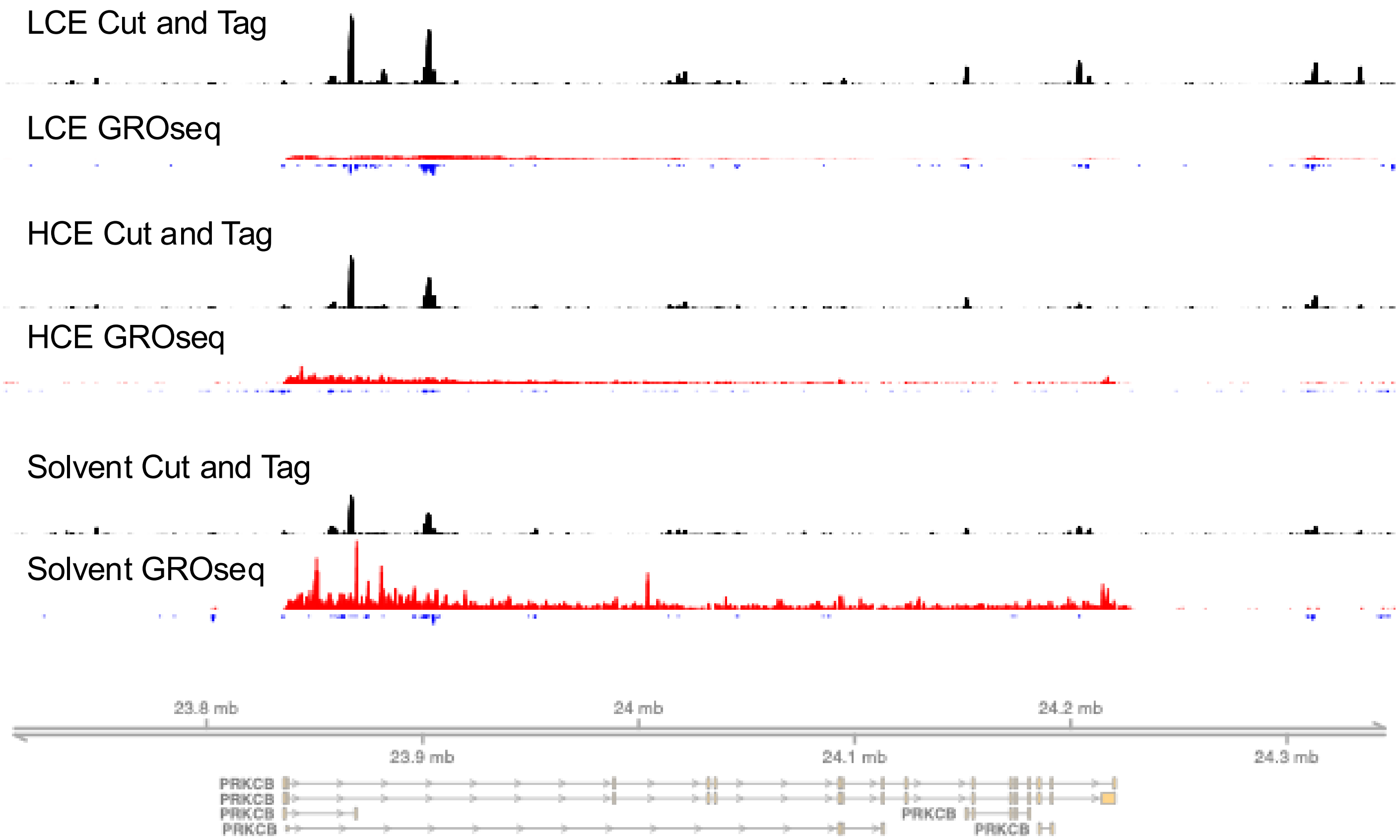

Figure S3: ***PRKCB*** **locus demonstrates differential effects of LCE vs. HCE.** Mithramycin suppresses expression of PRKCB by causing a loss of initiation with LCE (20 nmol/L; top) while HCE (100 nmol/L; middle) demonstrates reduced initiation coupled with impaired RNAPII processivity from TSS to TES. Data is the mean signal for biological replicates group scaled relative to the largest peak in control (bottom) following exposure to media for CUT&Tag solvent (n =3), LCE (n=3) or HCE (n = 3) and the associated average GROseq following exposure to media (n =3), LCE (n=3) or HCE (n = 3).

S4

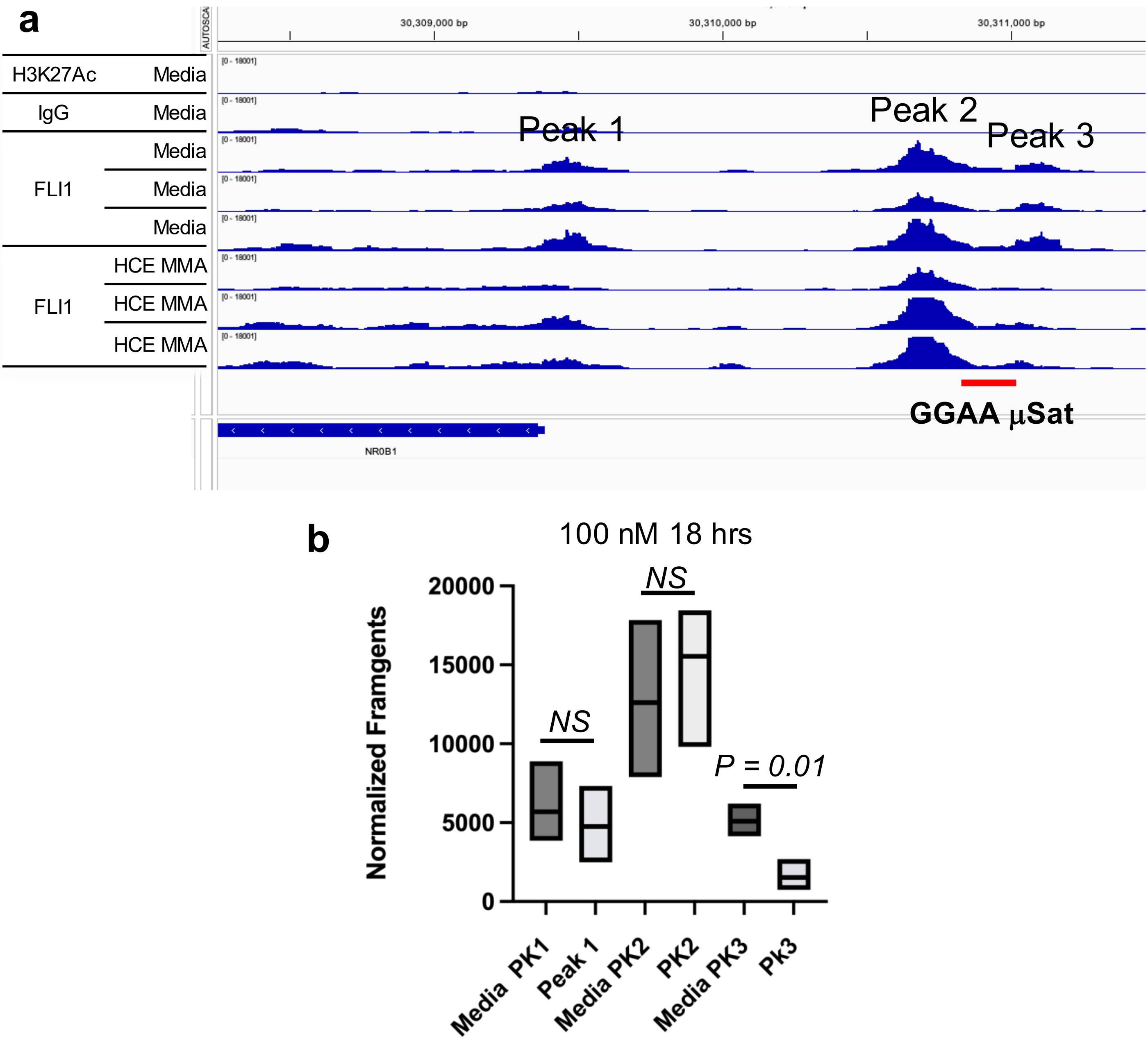

Figure S4: HCE reduces binding of EWS::FLI1 at the *NR0B1* locus with HCE. a. Reduction of EWS::FLI1 binding at the *NR0B1* locus as measured by CUT&Tag using a FLI1 antibody compared to H3K27ac positive and IgG negative control following exposure to HCE. 3 peaks are observed around the GGAA microsatellite and **b.** Quantitated as normalized fragment. Counts on the IGV. Box plots show minimum to maximum value and the mean for 3 independent biological replicates for each condition (two tailed t-test: *P in figure*)(See source data for full statistics)(Pk = Peak labeled in figure).

S5

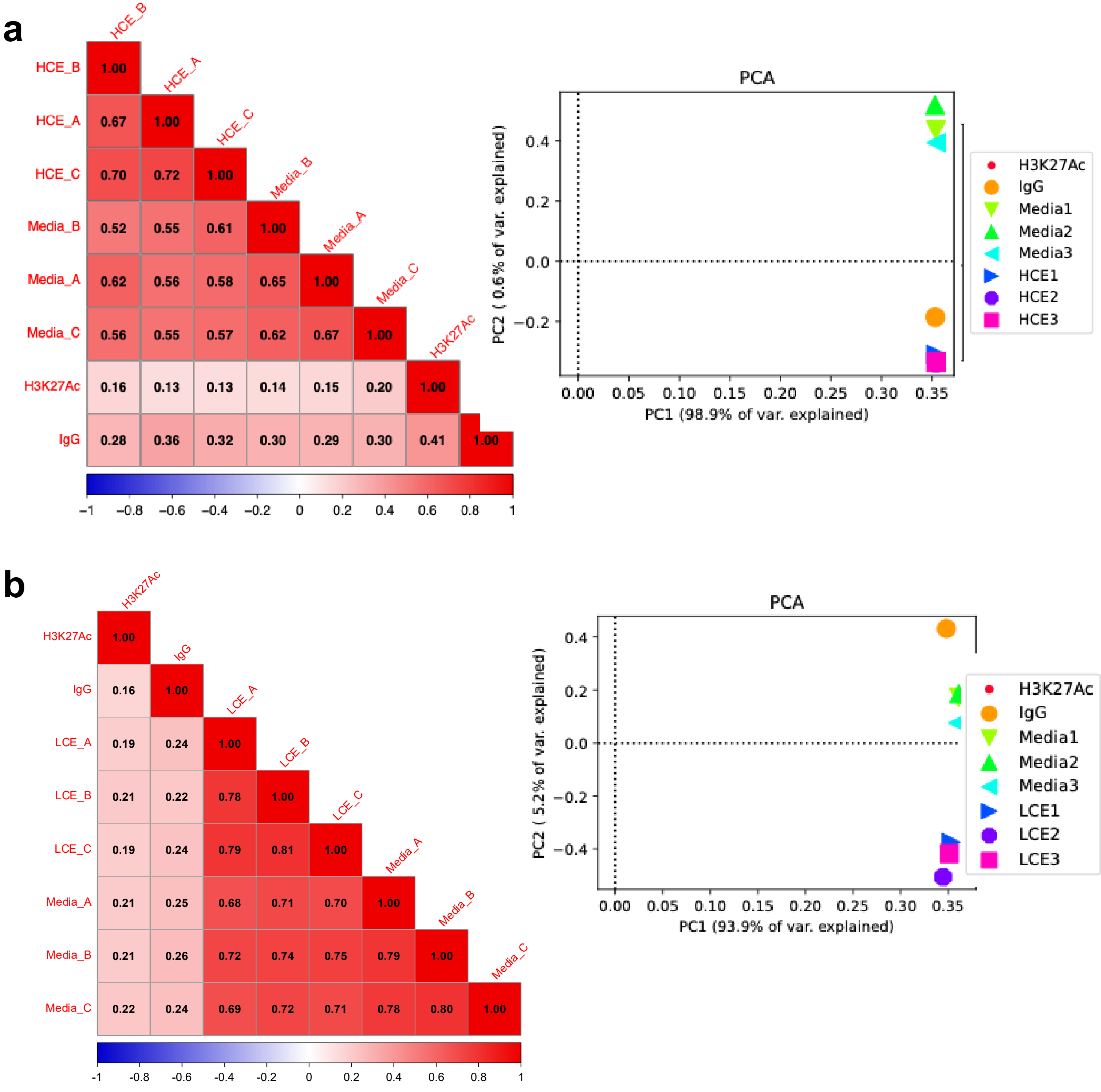

Fig. S5: Quality Control for Cut and Tag: Correlation matrix and PCA for **a.** HCE and **b.** LCE CUT&Tag showing consistency among treatment groups and replicates. Please note Media 3 is actually PBS solvent control (see methods) which shows good correlation with media.

S6

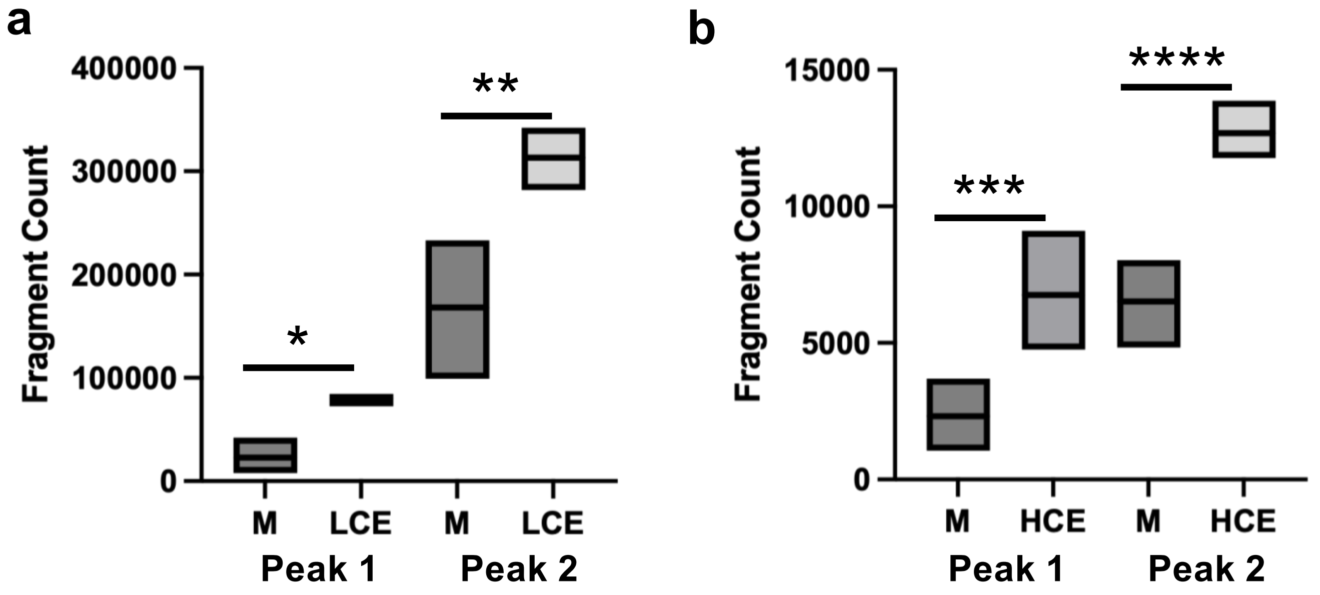

Figure S6: Quantitation of gain in occupancy of EWS::FLI1 at the *RCOR1* locus with LCE and HCE mithramycin. Quantitation of the peaks at the RCOR1 locus shown in Fig. 4f following exposure to **a.** LCE (n=3) or **b.** HCE (n=3) compared to media (n=3 for each). Data represents normalized fragment count. Box plot shows minimum to maximum value and the mean for three independent biological replicates for each condition. (two tailed t-test, **P* = 0.0062, **P = 0.0269, ****P* = 0.04, *****P* = 0.0052). *See* source data for full statistics.

S7

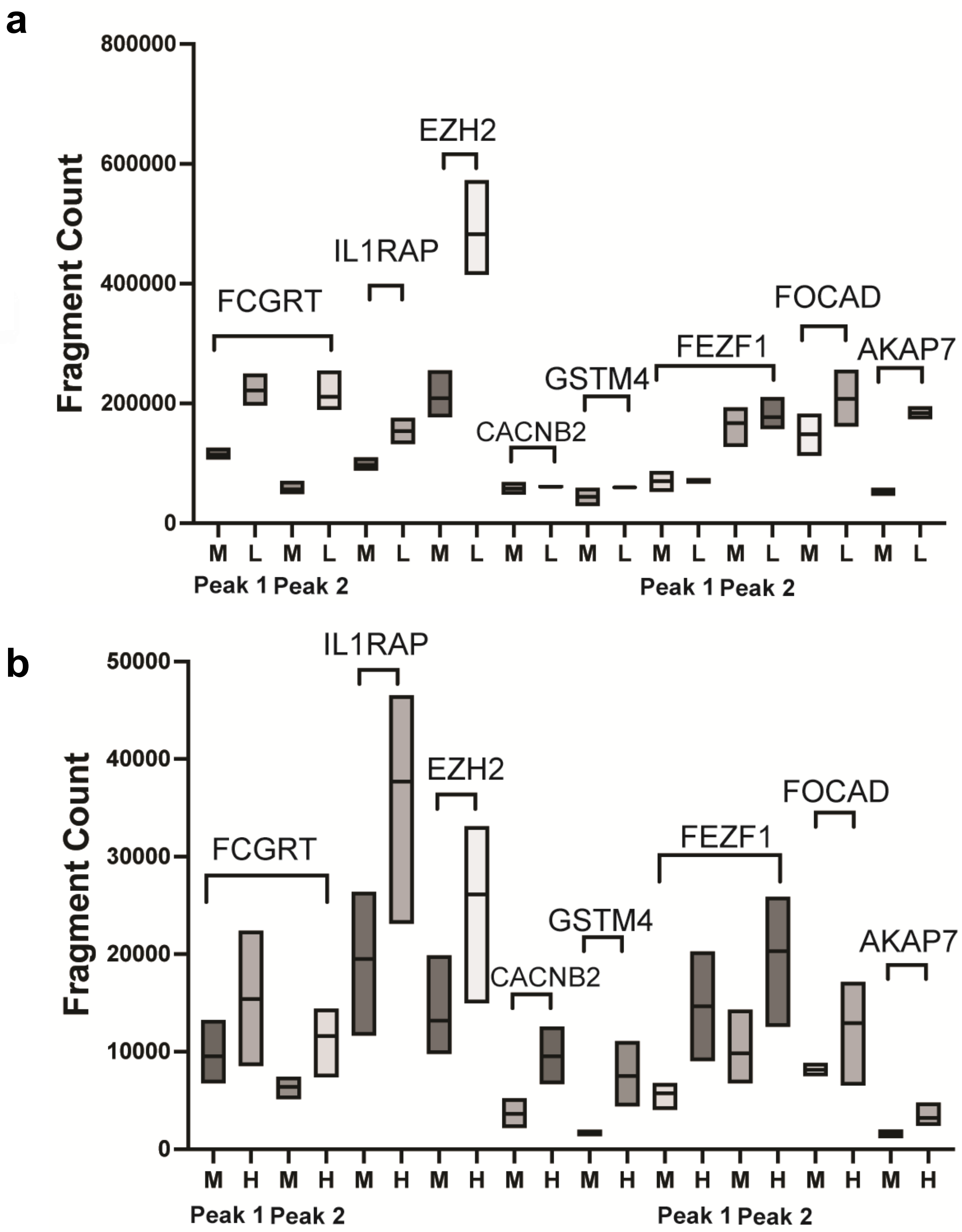

Figure S7: Quantitation of gain in occupancy of EWS::FLI1 at a panel of GGAA microsatellite driven downstream targets with LCE and HCE mithramycin. Quantitation of the peaks at the described loci comparing media (M, N=3) to exposure to **a.** LCE (L, n=3) ((1-way ANOVA $P < 0.0001$) or **b.** Media (n=3) to HCE (n=3)(1-way ANOVA $P < 0.0001$). Data represents normalized fragment count. Box plot shows minimum to maximum value and the mean for three independent biological replicates for each condition.

S8a

Rank	Motif	Name	*P-value*	Targets (no.)	Targets (%)	Back-ground (#)	Back-ground (%)
1	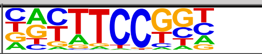	Fli1(ETS)/CD8-FLI-ChIP-Seq(GSE20898)/Homer	1e-3556	13032	37.11%	3757.3	10.99%
2	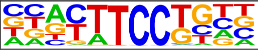	Etv2(ETS)/ES-ER71-ChIP-Seq(GSE59402)/Homer	1e-3319	12140	34.57%	3452.6	10.10%
3	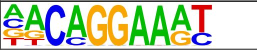	EWS:FLI1-fusion(ETS)/SK_N_MC-EWS:FLI1-ChIP-Seq(SRA014231)/Homer	1e-3244	8997	25.62%	1911.6	5.59%
4	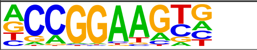	ETV4(ETS)/HepG2-ETV4-ChIP-Seq(ENCODE)/Homer	1e-3176	12509	35.62%	3786.7	11.08%
5	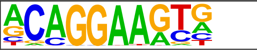	ERG(ETS)/VCaP-ERG-ChIP-Seq(GSE14097)/Homer	1e-3079	16049	45.70%	6156.9	18.01%
6	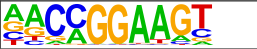	ETV1(ETS)/GIST48-ETV1-ChIP-Seq(GSE22441)/Homer	1e-2974	14143	40.27%	4993.8	14.61%
7	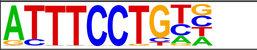	EWS:ERG-fusion(ETS)/CADO_ES1-EWS:ERG-ChIP-Seq(SRA014231)/Homer	1e-2834	10078	28.70%	2725	7.97%
8	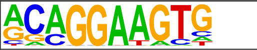	ETS1(ETS)/Jurkat-ETS1-ChIP-Seq(GSE17954)/Homer	1e-2828	12065	34.35%	3849.2	11.26%
9	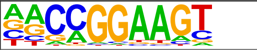	GABPA(ETS)/Jurkat-GABPa-ChIP-Seq(GSE17954)/Homer	1e-2650	10649	30.32%	3193.7	9.34%
10	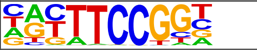	Elk4(ETS)/Hela-Elk4-ChIP-Seq(GSE31477)/Homer	1e-2316	6917	19.70%	1537.2	4.50%
11	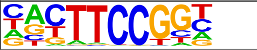	Elk1(ETS)/Hela-Elk1-ChIP-Seq(GSE31477)/Homer	1e-2291	7063	20.11%	1620.9	4.74%
12	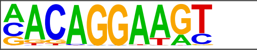	Ets1-distal(ETS)/CD4+-PolII-ChIP-Seq(Barski_et_al.)/Homer	1e-1935	5295	15.08%	1058	3.10%
13	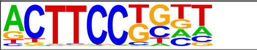	Elf4(ETS)/BMDM-Elf4-ChIP-Seq(GSE88699)/Homer	1e-1799	10191	29.02%	3817.7	11.17%
14	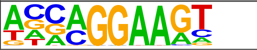	EHF(ETS)/LoVo-EHF-ChIP-Seq(GSE49402)/Homer	1e-1668	11860	33.77%	5117	14.97%
15	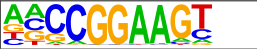	ELF1(ETS)/Jurkat-ELF1-ChIP-Seq(SRA014231)/Homer	1e-1457	5656	16.11%	1554.5	4.55%
16	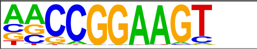	ETS(ETS)/Promoter/Homer	1e-1415	4402	12.53%	985.1	2.88%
17	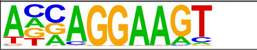	ELF3(ETS)/PDAC-ELF3-ChIP-Seq(GSE64557)/Homer	1e-1405	7684	21.88%	2741.1	8.02%
18	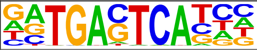	Jun-AP1(bZIP)/K562-cJun-ChIP-Seq(GSE31477)/Homer	1e-1394	3353	9.55%	568.1	1.66%
19	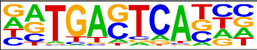	Fosl2(bZIP)/3T3L1-Fosl2-ChIP-Seq(GSE56872)/Homer	1e-1389	4046	11.52%	847.8	2.48%
20	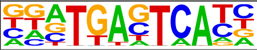	Fos(bZIP)/TSC-Fos-ChIP-Seq(GSE110950)/Homer	1e-1334	5739	16.34%	1706.1	4.99%
21	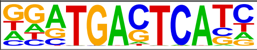	Fra2(bZIP)/Striatum-Fra2-ChIP-Seq(GSE43429)/Homer	1e-1334	4965	14.14%	1308.4	3.83%
22	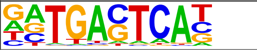	JunB(bZIP)/DendriticCells-Junb-ChIP-Seq(GSE36099)/Homer	1e-1315	5469	15.57%	1581.4	4.63%
23	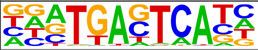	Fra1(bZIP)/BT549-Fra1-ChIP-Seq(GSE46166)/Homer	1e-1309	5442	15.50%	1572.5	4.60%
24	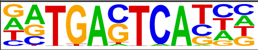	Atf3(bZIP)/GBM-ATF3-ChIP-Seq(GSE33912)/Homer	1e-1250	6039	17.20%	1954.8	5.72%
25	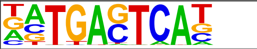	BATF(bZIP)/Th17-BATF-ChIP-Seq(GSE39756)/Homer	1e-1201	5921	16.86%	1940	5.68%
26	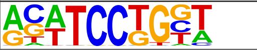	SPDEF(ETS)/VCaP-SPDEF-ChIP-Seq(SRA014231)/Homer	1e-1182	8437	24.02%	3519.5	10.30%
27	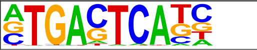	AP-1(bZIP)/ThioMac-PU.1-ChIP-Seq(GSE21512)/Homer	1e-1107	6243	17.78%	2235.7	6.54%
28	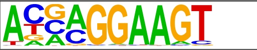	ELF5(ETS)/T47D-ELF5-ChIP-Seq(GSE30407)/Homer	1e-940	6720	19.13%	2757.2	8.07%
29	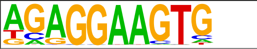	PU.1(ETS)/ThioMac-PU.1-ChIP-Seq(GSE21512)/Homer	1e-696	4820	13.72%	1911.3	5.59%
30	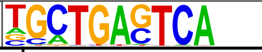	Bach2(bZIP)/OCILy7-Bach2-ChIP-Seq(GSE44420)/Homer	1e-522	2042	5.81%	543.9	1.59%
37	RCAGGATGTGGT	ETS:RUNX(ETS,Runt)/Jurkat-RUNX1-ChIP-Seq(GSE17954)/Homer	1.00E-162	858	2.44%	281.4	0.82%
41	GGCCCCGCCCCC	Sp1(Zf)/Promoter/Homer	1.00E-152	1399	3.98%	619.1	1.81%
65	VGCCATAAAA	Hoxd11(Homeobox)/ChickenMSG-Hoxd11.Flag-ChIP-Seq(GSE86088)/Homer	1.00E-77	10280	29.27%	8501.5	24.87%
66	NCYAATAAAA	Hoxd13(Homeobox)/ChickenMSG-Hoxd13.Flag-ChIP-Seq(GSE86088)/Homer	1.00E-77	7129	20.30%	5636.7	16.49%
74	GGCGGGAARN	E2F6(E2F)/Hela-E2F6-ChIP-Seq(GSE31477)/Homer	1.00E-65	2225	6.34%	1486.4	4.35%
80	GCTGTGGTTW	RUNX-AML(Runt)/CD4+-PolII-ChIP-Seq(Barski_et_al.)/Homer	1.00E-60	2806	7.99%	1991.6	5.83%

S8b

Ρανκ	Μοτιφ	Π−ϖαλυε	λογ Π−πϖαλυε	% οφ Ταργετσ	% οφ Βαχκγρουνδ	ΣΤΔ(Βγ ΣΤΔ)	Βεστ Ματχη/Δεταιλσ
1	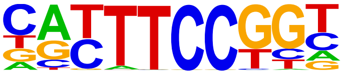	1ε−3722	−8.571ε+03	30.61%	7.13%	45.7βπ (63.9βπ)	Ελκ1(ΕΤΣ)/Ηελα−Ελκ1−ΧηΙΠ−Σεθ(ΓΣΕ31477)/Ηομερ(0.973) Μορε Ινφορματιον | Σιμιλαρ Μοτιφσ Φουνδ
2	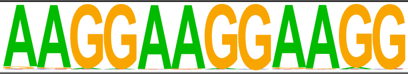	1ε−2317	−5.337ε+03	8.33%	0.55%	60.3βπ (101.6βπ)	ΕΩΣΡ1−ΦΛΙ1/ΜΑ0149.1/ϑασπαρ(0.825) Μορε Ινφορματιον | Σιμιλαρ Μοτιφσ Φουνδ
3	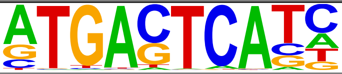	1ε−1483	−3.415ε+03	10.01%	1.72%	48.8βπ (61.0βπ)	ΦΟΣ::ϑΥΝΒ/ΜΑ1134.1/ϑασπαρ(0.991) Μορε Ινφορματιον | Σιμιλαρ Μοτιφσ Φουνδ
4	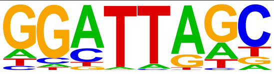	1ε−239	−5.521ε+02	13.42%	8.18%	53.8βπ (64.2βπ)	ΧΡΞ(Ηομεοβοξ)/Ρετινα−Χρξ−ΧηΙΠ−Σεθ(ΓΣΕ20012)/Ηομερ(0.970) Μορε Ινφορματιον | Σιμιλαρ Μοτιφσ Φουνδ
5	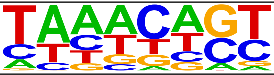	1ε−219	−5.054ε+02	42.45%	34.30%	56.0βπ (64.0βπ)	ΦΟΞΛ1/ΜΑ0033.2/ϑασπαρ(0.703) Μορε Ινφορματιον | Σιμιλαρ Μοτιφσ Φουνδ
6	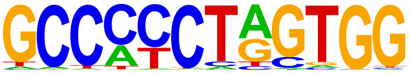	1ε−210	−4.854ε+02	2.21%	0.57%	44.4βπ (59.8βπ)	ΒΟΡΙΣ(Ζφ)/Κ562−ΧΤΧΦΛ−ΧηΙΠ−Σεθ(ΓΣΕ32465)/Ηομερ(0.923) Μορε Ινφορματιον | Σιμιλαρ Μοτιφσ Φουνδ
7	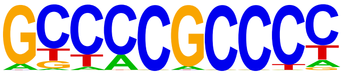	1ε−203	−4.688ε+02	4.54%	1.92%	53.7βπ (57.5βπ)	Σπ2(Ζφ)/ΗΕΚ293−Σπ2.εΓΦΠ−ΧηΙΠ−Σεθ(Ενχοδε)/Ηομερ(0.968) Μορε Ινφορματιον | Σιμιλαρ Μοτιφσ Φουνδ
8	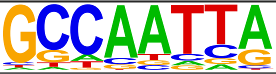	1ε−156	−3.605ε+02	38.09%	31.37%	56.4βπ (62.1βπ)	ΜΣΞ2/ΜΑ0708.1/ϑασπαρ(0.848) Μορε Ινφορματιον | Σιμιλαρ Μοτιφσ Φουνδ
9	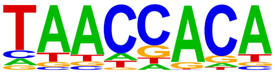	1ε−114	−2.642ε+02	7.59%	4.78%	55.8βπ (58.9βπ)	ΡΥΝΞ−ΑΜΛ(Ρυντ)/ΧΔ4+−ΠολΙΙ−ΧηΙΠ−Σεθ(Βαρσκι_ετ_αλ.)/Ηομερ(0.911) Μορε Ινφορματιον | Σιμιλαρ Μοτιφσ Φουνδ
10	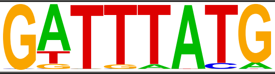	1ε−110	−2.533ε+02	8.12%	5.25%	57.1βπ (60.9βπ)	ΜΕΙΣ2(ϖαρ.2)/ΜΑ1640.1/ϑασπαρ(0.923) Μορε Ινφορματιον | Σιμιλαρ Μοτιφσ Φουνδ
11	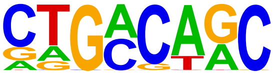	1ε−102	−2.369ε+02	22.97%	18.37%	55.3βπ (65.2βπ)	ΗΙΧ1(Ζφ)/Τρεγ−ΖΒΤΒ29−ΧηΙΠ−Σεθ(ΓΣΕ99889)/Ηομερ(0.818) Μορε Ινφορματιον | Σιμιλαρ Μοτιφσ Φουνδ
12	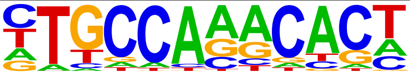	1ε−87	−2.021ε+02	15.00%	11.47%	55.0βπ (61.9βπ)	ΝΦ1−ηαλφσιτε(ΧΤΦ)/ΛΝΧαΠ−ΝΦ1−ΧηΙΠ−Σεθ(Υνπυβλισηεδ)/Ηομερ(0.764) Μορε Ινφορματιον | Σιμιλαρ Μοτιφσ Φουνδ
13	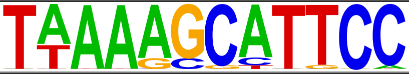	1ε−85	−1.964ε+02	0.21%	0.01%	51.7βπ (35.3βπ)	ΤΕΑΔ3/ΜΑ0808.1/ϑασπαρ(0.698) Μορε Ινφορματιον | Σιμιλαρ Μοτιφσ Φουνδ
14	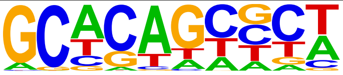	1ε−78	−1.818ε+02	23.87%	19.77%	53.6βπ (64.7βπ)	ΖΝΦ416(Ζφ)/ΗΕΚ293−ΖΝΦ416.ΓΦΠ−ΧηΙΠ−Σεθ(ΓΣΕ58341)/Ηομερ(0.660) Μορε Ινφορματιον | Σιμιλαρ Μοτιφσ Φουνδ
15	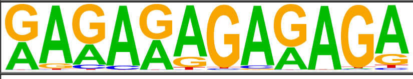	1ε−78	−1.802ε+02	2.96%	1.56%	57.3βπ (75.0βπ)	ΠΡΔΜ1/ΜΑ0508.3/ϑασπαρ(0.741) Μορε Ινφορματιον | Σιμιλαρ Μοτιφσ Φουνδ
16	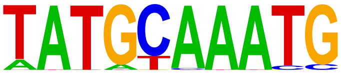	1ε−55	−1.279ε+02	0.76%	0.24%	54.4βπ (54.3βπ)	Οχτ4(ΠΟΥ,Ηομεοβοξ)/μΕΣ−Οχτ4−ΧηΙΠ−Σεθ(ΓΣΕ11431)/Ηομερ(0.932) Μορε Ινφορματιον | Σιμιλαρ Μοτιφσ Φουνδ
17	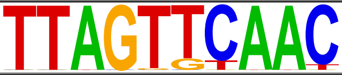	1ε−22	−5.259ε+01	0.28%	0.08%	50.5βπ (52.0βπ)	ςΔΡ/ΜΑ0693.2/ϑασπαρ(0.687) Μορε Ινφορματιον | Σιμιλαρ Μοτιφσ Φουνδ

Figure S8: Mithramycin alters EWS::FLI1 binding to chromatin and favors the ETS factor GGAA motif. a. Known or **b,** *De Novo* Homer motif analysis of CUT&Tag sequencing data obtained after exposure to LCE (Comparison of media (n=3) to LCE (n=3).

S9a

Rank & Motif		Name	*P-value*	Targets (no.)	Targets (%)	Back-ground (#)	Back-ground (%)
1	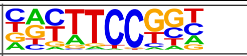	Fli1(ETS)/CD8-FLI-ChIP-Seq(GSE20898)/Homer	1e-903	3627	34.63%	4216.8	10.89%
2	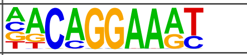	EWS:FLI1-fusion(ETS)/SK_N_MC-EWS:FLI1-ChIP-Seq(SRA014231)/Homer	1e-879	2565	24.49%	2167.9	5.60%
3	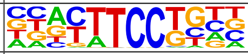	Etv2(ETS)/ES-ER71-ChIP-Seq(GSE59402)/Homer	1e-864	3420	32.65%	3896.1	10.06%
4	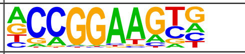	ETV4(ETS)/HepG2-ETV4-ChIP-Seq(ENCODE)/Homer	1e-795	3462	33.05%	4234.5	10.93%
5	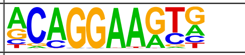	ERG(ETS)/VCaP-ERG-ChIP-Seq(GSE14097)/Homer	1e-781	4532	43.26%	6951.3	17.95%
6	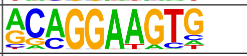	ETS1(ETS)/Jurkat-ETS1-ChIP-Seq(GSE17954)/Homer	1e-722	3376	32.23%	4321.6	11.16%
7	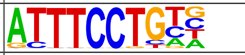	EWS:ERG-fusion(ETS)/CADO_ES1-EWS:ERG-ChIP-Seq(SRA014231)/Homer	1e-696	2835	27.06%	3217.6	8.31%
8	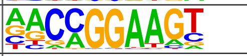	ETV1(ETS)/GIST48-ETV1-ChIP-Seq(GSE22441)/Homer	1e-695	3883	37.07%	5671.5	14.64%
9	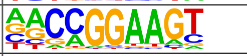	GABPA(ETS)/Jurkat-GABPa-ChIP-Seq(GSE17954)/Homer	1e-679	2939	28.06%	3505	9.05%
10	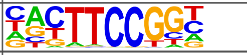	Elk1(ETS)/Hela-Elk1-ChIP-Seq(GSE31477)/Homer	1e-656	1959	18.70%	1648.2	4.26%
11	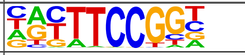	Elk4(ETS)/Hela-Elk4-ChIP-Seq(GSE31477)/Homer	1e-646	1935	18.47%	1630.2	4.21%
12	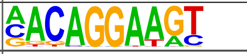	Ets1-distal(ETS)/CD4+-PolII-ChIP-Seq(Barski_et_al.)/Homer	1e-517	1498	14.30%	1200.7	3.10%
13	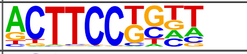	Elf4(ETS)/BMDM-Elf4-ChIP-Seq(GSE88699)/Homer	1e-399	2768	26.42%	4362.2	11.26%
14	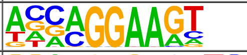	EHF(ETS)/LoVo-EHF-ChIP-Seq(GSE49402)/Homer	1e-368	3276	31.27%	5908	15.25%
15	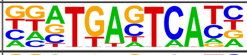	Fos(bZIP)/TSC-Fos-ChIP-Seq(GSE110950)/Homer	1e-364	1687	16.11%	2012.8	5.20%
16	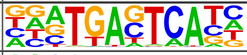	Fra1(bZIP)/BT549-Fra1-ChIP-Seq(GSE46166)/Homer	1e-361	1610	15.37%	1862.1	4.81%
17	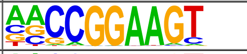	ETS(ETS)/Promoter/Homer	1e-359	1171	11.18%	1036.2	2.68%
18	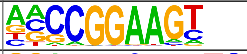	ELF1(ETS)/Jurkat-ELF1-ChIP-Seq(SRA014231)/Homer	1e-355	1489	14.21%	1639.7	4.23%
19	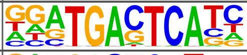	Fra2(bZIP)/Striatum-Fra2-ChIP-Seq(GSE43429)/Homer	1e-354	1450	13.84%	1567.5	4.05%
20	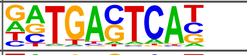	JunB(bZIP)/DendriticCells-Junb-ChIP-Seq(GSE36099)/Homer	1e-349	1580	15.08%	1844.7	4.76%
21	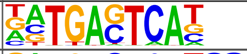	BATF(bZIP)/Th17-BATF-ChIP-Seq(GSE39756)/Homer	1e-343	1745	16.66%	2217.6	5.73%
22	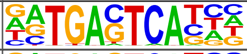	Atf3(bZIP)/GBM-ATF3-ChIP-Seq(GSE33912)/Homer	1e-340	1772	16.92%	2292.7	5.92%
23	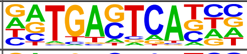	Fosl2(bZIP)/3T3L1-Fosl2-ChIP-Seq(GSE56872)/Homer	1e-339	1147	10.95%	1047.4	2.70%
24	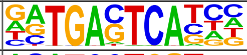	Jun-AP1(bZIP)/K562-cJun-ChIP-Seq(GSE31477)/Homer	1e-332	928	8.86%	701.4	1.81%
25	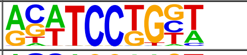	SPDEF(ETS)/VCaP-SPDEF-ChIP-Seq(SRA014231)/Homer	1e-326	2466	23.54%	4019.2	10.38%
26	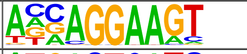	ELF3(ETS)/PDAC-ELF3-ChIP-Seq(GSE64557)/Homer	1e-314	2149	20.52%	3294.3	8.51%
27	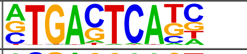	AP-1(bZIP)/ThioMac-PU.1-ChIP-Seq(GSE21512)/Homer	1.00E-300	1828	17.45%	2602.5	6.72%
28	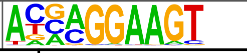	ELF5(ETS)/T47D-ELF5-ChIP-Seq(GSE30407)/Homer	1.00E-214	1852	17.68%	3159.7	8.16%
29	AGAGGAAGTG	PU.1(ETS)/ThioMac-PU.1-ChIP-Seq(GSE21512)/Homer	1.00E-162	1303	12.44%	2112	5.45%
38	RCAGGATGTGGT	ETS:RUNX(ETS,Runt)/Jurkat-RUNX1-ChIP-Seq(GSE17954)/Homer	1.00E-49	245	2.34%	295.6	0.76%
39	VGCCATAAAA	Hoxd11(Homeobox)/ChickenMSG-Hoxd11.Flag-ChIP-Seq(GSE86088)/Homer	1.00E-45	3356	32.04%	9996.3	25.81%
40	NCYAATAAAA	Hoxd13(Homeobox)/ChickenMSG-Hoxd13.Flag-ChIP-Seq(GSE86088)/Homer	1.00E-45	2385	22.77%	6713.3	17.33%
62	GGCMATGAAA	Hoxd10(Homeobox)/ChickenMSG-Hoxd10.Flag-ChIP-Seq(GSE86088)/Homer	1.00E-29	1614	15.41%	4537.5	11.71%
118	GCTGTGGTTW	RUNX-AML(Runt)/CD4+-PolII-ChIP-Seq(Barski_et_al.)/Homer	1.00E-12	825	7.88%	2371	6.12%
119	AAACCACARM	RUNX1(Runt)/Jurkat-RUNX1-ChIP-Seq(GSE29180)/Homer	1.00E-12	1167	11.14%	3519.8	9.09%
120	GGCGGGAARN	E2F6(E2F)/Hela-E2F6-ChIP-Seq(GSE31477)/Homer	1.00E-11	559	5.34%	1522.5	3.93%

S9b

Ρανκ	Μοτιφ	Π-ϖαλυε	λογ Π-πϖαλυε	% οφ Ταργετσ	% οφ Βαχκγρουνδ	ΣΤΔ(Βγ ΣΤΔ)	Βεστ Ματχη/Δεταιλσ
1	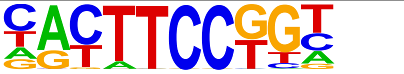	1ε-972	-2.239ε+03	28.07%	6.75%	47.3βπ (65.6βπ)	ΕΤς1(ΕΤΣ)/ΓΙΣΤ48-ΕΤς1-ΧηΙΠ-Σεθ(ΓΣΕ22441)/Ηομερ(0.970) Μορε Ινφορματιον | Σιμιλαρ Μοτιφσ Φουνδ
2	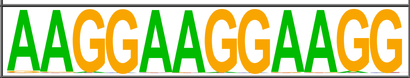	1ε-624	-1.439ε+03	7.77%	0.55%	60.5βπ (97.0βπ)	ΕΩΣΡ1-ΦΛΙ1/ΜΑ0149.1/ϑασπαρ(0.825) Μορε Ινφορματιον | Σιμιλαρ Μοτιφσ Φουνδ
3	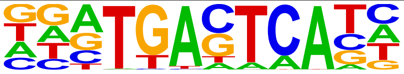	1ε-389	-8.968ε+02	11.46%	2.61%	51.2βπ (60.5βπ)	Φρα1(βΖΙΠ)/ΒΤ549-Φρα1-ΧηΙΠ-Σεθ(ΓΣΕ46166)/Ηομερ(0.995) Μορε Ινφορματιον | Σιμιλαρ Μοτιφσ Φουνδ
4	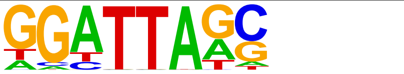	1ε-84	-1.942ε+02	18.81%	12.15%	55.9βπ (63.6βπ)	ΧΡΞ(Ηομεοβοξ)/Ρετινα-Χρξ-ΧηΙΠ-Σεθ(ΓΣΕ20012)/Ηομερ(0.981) Μορε Ινφορματιον | Σιμιλαρ Μοτιφσ Φουνδ
5	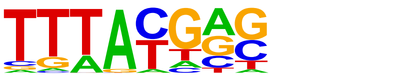	1ε-72	-1.667ε+02	22.77%	15.98%	56.7βπ (61.9βπ)	ΧΔΞ4(Ηομεοβοξ)/ΖεβραφισηΕμβρψοσ-Χδξ4.Μψχ-ΧηΙΠ-Σεθ(ΓΣΕ48254)/Ηομερ(0.923) Μορε Ινφορματιον | Σιμιλαρ Μοτιφσ Φουνδ
6	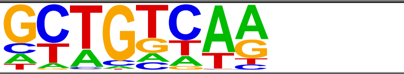	1ε-69	-1.604ε+02	27.60%	20.36%	55.3βπ (64.8βπ)	ΜΕΙΣ2/ΜΑ0774.1/ϑασπαρ(0.891) Μορε Ινφορματιον | Σιμιλαρ Μοτιφσ Φουνδ
7	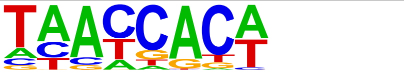	1ε-61	-1.423ε+02	30.02%	22.96%	55.8βπ (60.7βπ)	ΡΥΝΞ-ΑΜΛ(Ρυντ)/ΧΔ4+-ΠολΙΙ-ΧηΙΠ-Σεθ(Βαρσκι_ετ_αλ.)/Ηομερ(0.853) Μορε Ινφορματιον | Σιμιλαρ Μοτιφσ Φουνδ
8	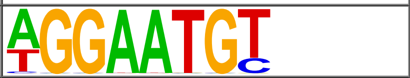	1ε-46	-1.061ε+02	7.67%	4.49%	55.4βπ (65.6βπ)	ΤΕΑΔ2/ΜΑ1121.1/ϑασπαρ(0.969) Μορε Ινφορματιον | Σιμιλαρ Μοτιφσ Φουνδ
9	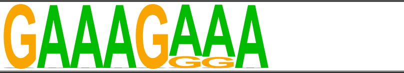	1ε-45	-1.041ε+02	12.63%	8.51%	57.0βπ (74.6βπ)	ΠΡΔΜ1/ΜΑ0508.3/ϑασπαρ(0.731) Μορε Ινφορματιον | Σιμιλαρ Μοτιφσ Φουνδ
10	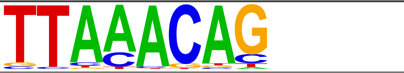	1ε-44	-1.020ε+02	24.44%	18.89%	57.3βπ (62.3βπ)	Φοξφ1/ΜΑ1606.1/ϑασπαρ(0.808) Μορε Ινφορματιον | Σιμιλαρ Μοτιφσ Φουνδ
11	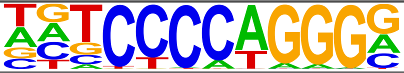	1ε-42	-9.760ε+01	5.65%	3.07%	53.3βπ (60.3βπ)	ΕΒΦ1/ΜΑ0154.4/ϑασπαρ(0.943) Μορε Ινφορματιον | Σιμιλαρ Μοτιφσ Φουνδ
12	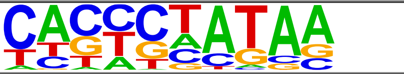	1ε-40	-9.291ε+01	1.46%	0.39%	55.6βπ (62.7βπ)	ΖΝΦ410/ΜΑ0752.1/ϑασπαρ(0.660) Μορε Ινφορματιον | Σιμιλαρ Μοτιφσ Φουνδ
13	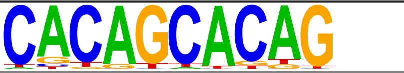	1ε-27	-6.417ε+01	8.64%	5.93%	55.3βπ (62.9βπ)	ΖΝΦ317/ΜΑ1593.1/ϑασπαρ(0.676) Μορε Ινφορματιον | Σιμιλαρ Μοτιφσ Φουνδ
14	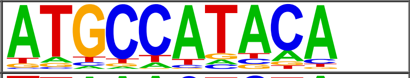	1ε-25	-5.948ε+01	8.34%	5.77%	55.8βπ (61.5βπ)	ΧΔΞ1/ΜΑ0878.2/ϑασπαρ(0.696) Μορε Ινφορματιον | Σιμιλαρ Μοτιφσ Φουνδ
15	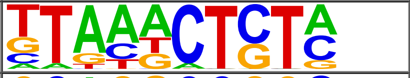	1ε-24	-5.572ε+01	9.55%	6.87%	55.6βπ (61.3βπ)	ΝΡ1Ι3/ΜΑ1534.1/ϑασπαρ(0.663) Μορε Ινφορματιον | Σιμιλαρ Μοτιφσ Φουνδ
16	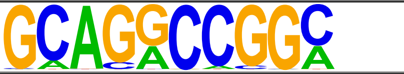	1ε-19	-4.462ε+01	0.91%	0.30%	55.0βπ (99.9βπ)	Ζφξ/ΜΑ0146.2/ϑασπαρ(0.610) Μορε Ινφορματιον | Σιμιλαρ Μοτιφσ Φουνδ
17	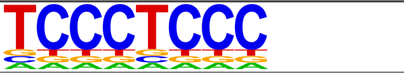	1ε-18	-4.308ε+01	5.32%	3.58%	58.0βπ (71.4βπ)	ΜΑΖ/ΜΑ1522.1/ϑασπαρ(0.799) Μορε Ινφορματιον | Σιμιλαρ Μοτιφσ Φουνδ
18	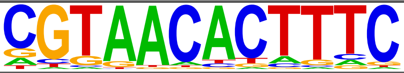	1ε-16	-3.876ε+01	0.22%	0.02%	58.1βπ (48.6βπ)	ΖΝΦ652/ΜΑ1657.1/ϑασπαρ(0.712) Μορε Ινφορματιον | Σιμιλαρ Μοτιφσ Φουνδ
19	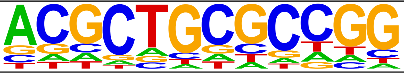	1ε-13	-3.214ε+01	0.11%	0.00%	54.1βπ (36.6βπ)	ΖΒΤΒ14/ΜΑ1650.1/ϑασπαρ(0.604) Μορε Ινφορματιον | Σιμιλαρ Μοτιφσ Φουνδ
20	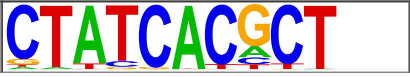	1ε-13	-3.187ε+01	0.51%	0.14%	59.1βπ (55.9βπ)	Αηρ::Αρντ/ΜΑ0006.1/ϑασπαρ(0.647) Μορε Ινφορματιον | Σιμιλαρ Μοτιφσ Φουνδ

S9c

Rank	Motif	P-value	log P-pvalue	% of Targets	% of Background	STD(Bg STD)
1	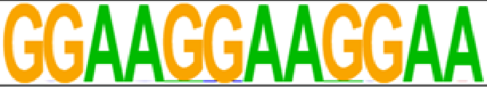	1e-1754	-4.040e+03	12.17%	0.63%	59.4bp (91.5bp)
2	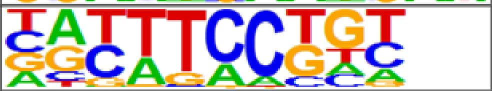	1e-1244	-2.865e+03	37.71%	13.67%	49.8bp (68.2bp)
3	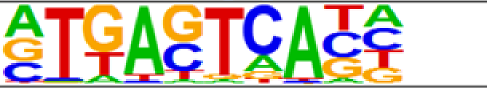	1e-866	-1.995e+03	12.32%	2.03%	49.6bp (60.6bp)
4	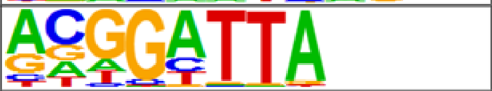	1e-125	-2.882e+02	21.26%	14.27%	55.3bp (65.3bp)
5	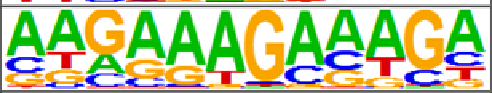	1e-110	-2.539e+02	5.02%	2.05%	57.0bp (74.3bp)
6	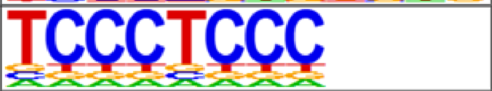	1e-94	-2.170e+02	7.09%	3.65%	58.3bp (83.0bp)
7	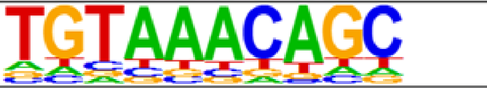	1e-87	-2.018e+02	30.19%	23.32%	55.7bp (62.7bp)
8	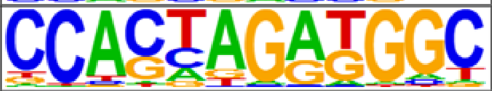	1e-84	-1.944e+02	1.69%	0.39%	49.3bp (59.2bp)
9	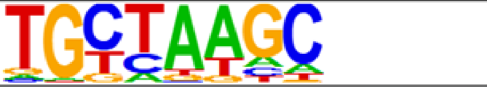	1e-83	-1.922e+02	24.50%	18.32%	55.8bp (65.8bp)
10	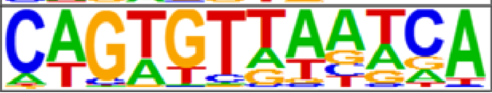	1e-76	-1.754e+02	17.42%	12.34%	54.2bp (62.0bp)

Figure S9: Mithramycin alters EWS::FLI1 binding to chromatin and favors the ETS factor GGAA motif. a. Known or **b.** *De Novo* Homer motif analysis of CUT&Tag sequencing data obtained after exposure to HCE (Comparison of media (n=3) to HCE (n=3). **c.** *De Novo* motif analysis of LCE (n=3) vs. HCE (n=3) demonstrating enrichment of the GGAA microsatellite enhancer that favors LCE.

S10

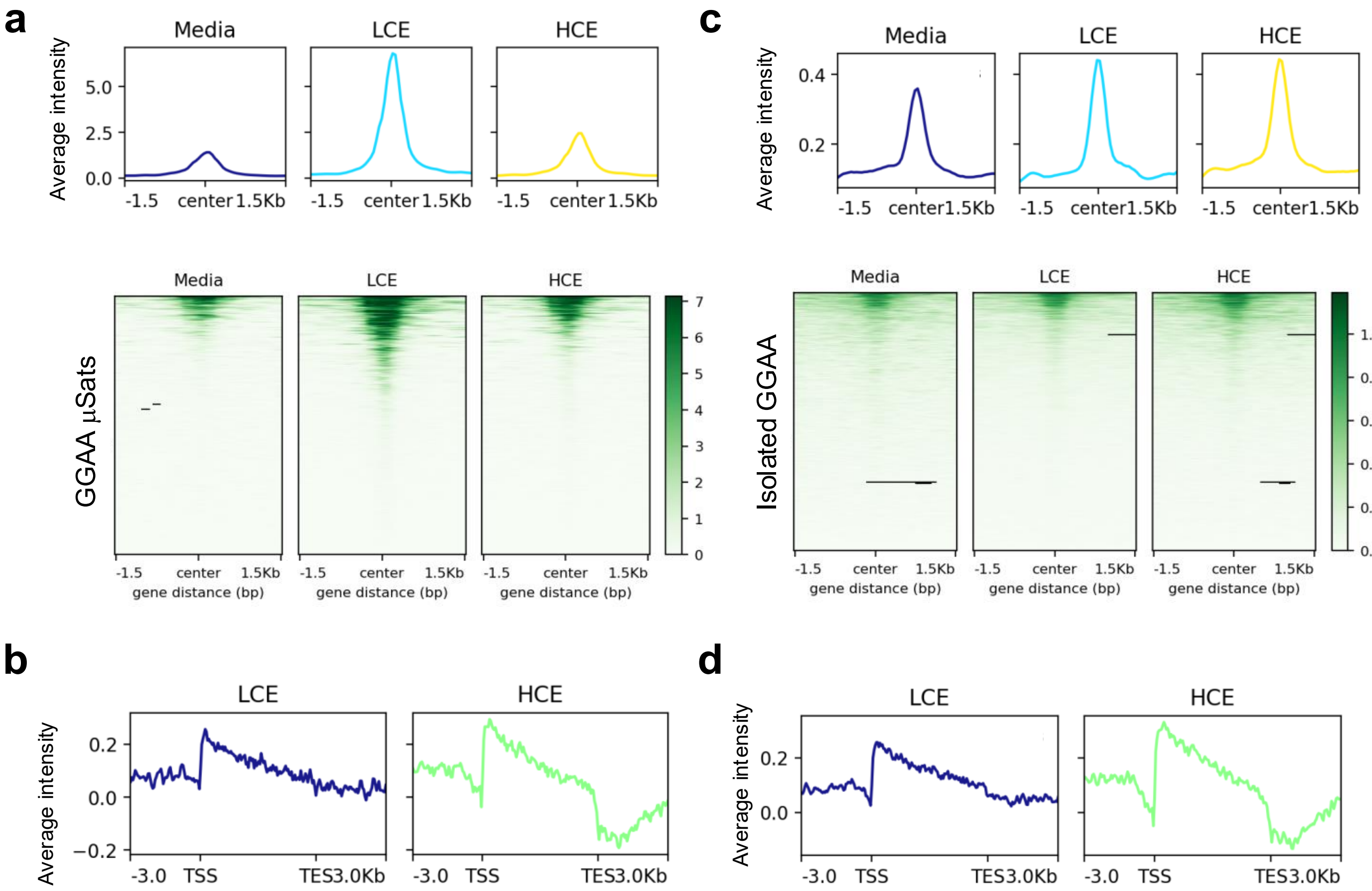

Figure S10: Gain in occupancy of EWS::FLI1 at GGAA microsatellites and isolated GGAAs with LCE and HCE mithramycin. a. Profile plot (top) of average normalized EWS::FLI1 CUT&Tag signal intensity and corresponding heat map (bottom) of EWS::FLI1 CUT&Tag signal centered on all GGAA microsatellites annotated by Homer to EWS::FLI1 repressed targets (n=487) following exposure to media (n=3), LCE (n=3), or HCE (n=3). Data demonstrates enrichment with LCE>HCE. **b.** Corresponding profile plot (bottom) of average Log_2FC (treated to control) GROseq signal intensity aligned from TSS to TES to these GGAA-microsatellite annotated EWS::FLI1 repressed targets showing increased nascent transcription initiation and productive transcription with LCE (n=2) and HCE (n=3). **c.** Profile plot (top) of average normalized EWS::FLI1 CUT&Tag signal intensity and corresponding heat map (bottom) of EWS::FLI1 CUT&Tag signal centered on isolated GGAA motifs annotated by Homer to EWS::FLI1 repressed targets (n=1060) following exposure to media, LCE, or HCE. Data demonstrates enrichment with LCE=HCE. **d.** Corresponding profile plot (bottom) of average Log_2FC (treated to control) GROseq signal intensity aligned from TSS to TES at these isolated GGAA-motifs annotated to EWS::FLI1 repressed targets showing induction of nascent transcription initiation and productive transcription with LCE and HCE.

S11

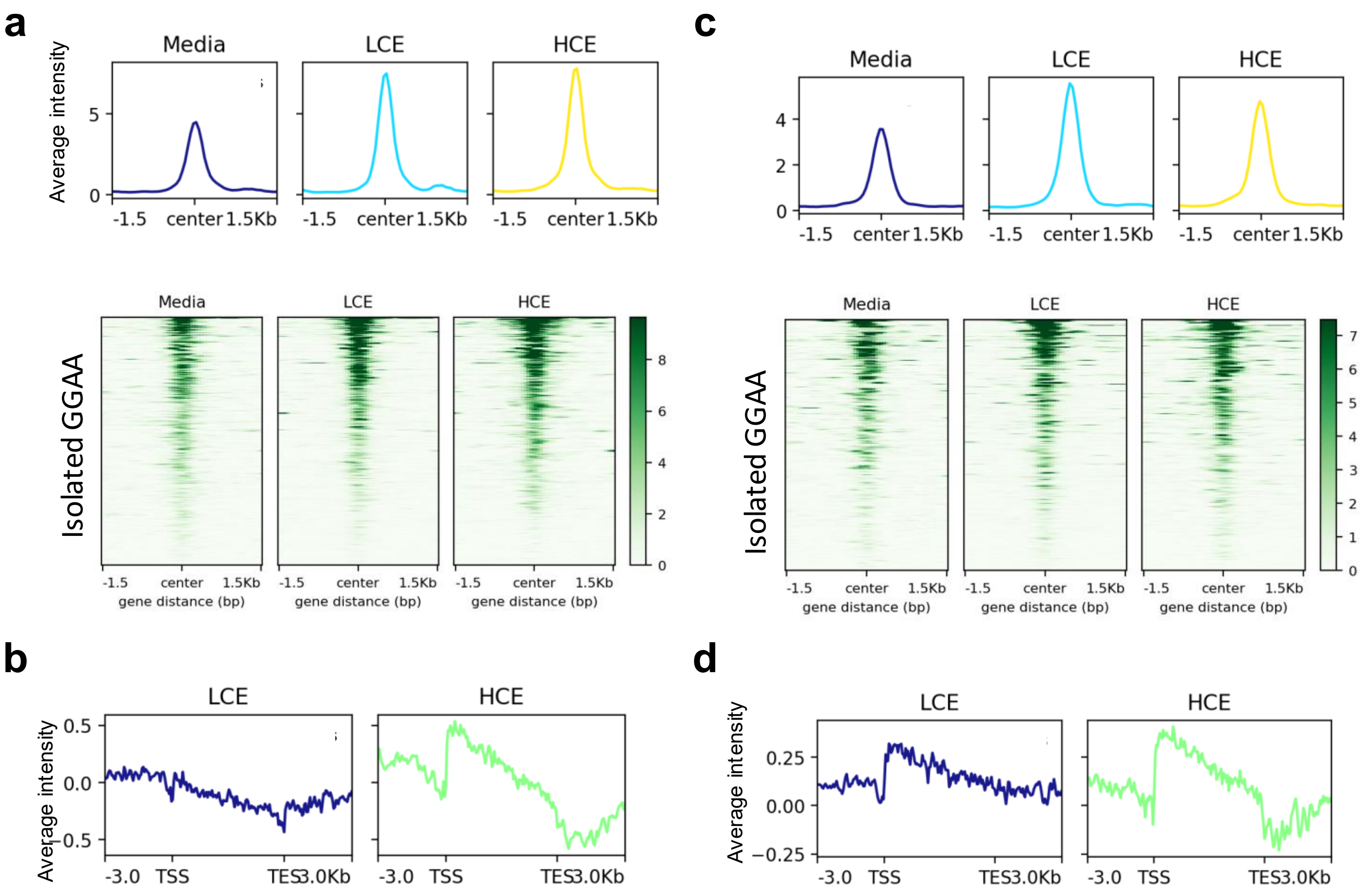

Figure S11: Gain in occupancy of EWS::FLI1 at isolated GGAA downstream target response elements with LCE and HCE mithramycin. a. Profile plot (top) of average normalized EWS::FLI1 CUT&Tag signal intensity and corresponding heat map (bottom) of EWS::FLI1 CUT&Tag signal centered on *differentially bound* isolated GGAA response elements annotated by Homer to EWS::FLI1 induced targets following exposure to media (n=3), LCE (n=3), or HCE (n=3). Data demonstrates enrichment with LCE=HCE. **b.** Corresponding profile plot (bottom) of average Log_2FC (treated to control) GROseq signal intensity aligned from TSS to TES to these differentially bound isolated GGAA response elements annotated to EWS::FLI1 induced targets showing decreased nascent transcription initiation and productive transcription with LCE (n=2). **c.** Profile plot (top) of average normalized EWS::FLI1 CUT&Tag signal intensity and corresponding heat map (bottom) of EWS::FLI1 CUT&Tag signal centered on *differentially bound* isolated GGAA motifs annotated by Homer to EWS::FLI1 repressed targets (n=1060) following exposure to media, LCE, or HCE. Data demonstrates enrichment with LCE=HCE. **d.** Corresponding profile plot (bottom) of average Log_2FC (treated to control) GROseq signal intensity aligned from TSS to TES at these isolated GGAA-motifs annotated to EWS::FLI1 repressed targets showing induction of nascent transcription initiation and productive transcription with LCE and HCE.

S12

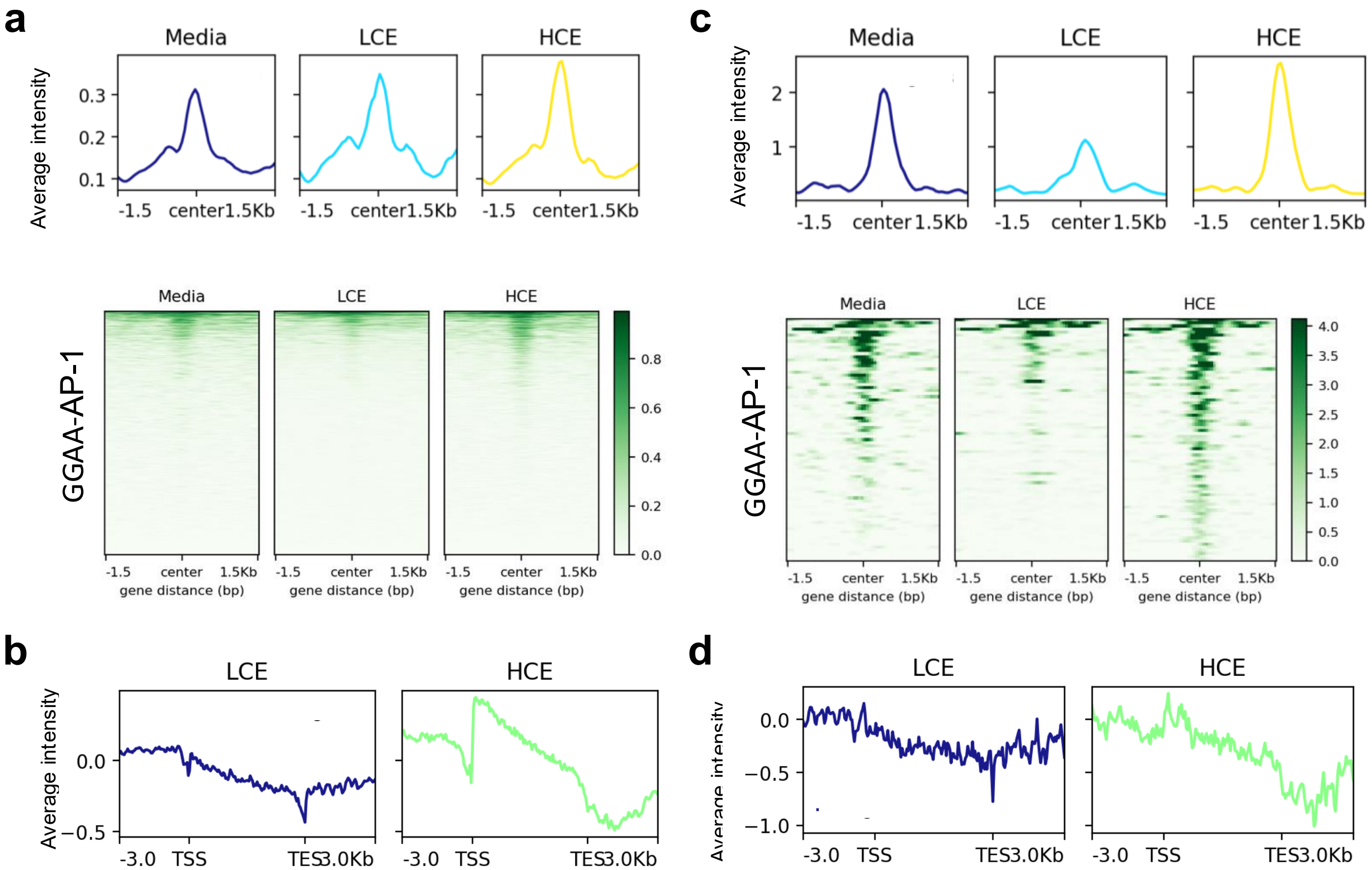

Figure S12: Loss of occupancy of EWS::FLI1 at tandem GGAA-AP-1 sites with LCE. Profile plot (top) of average normalized EWS::FLI1 CUT&Tag signal intensity and corresponding heat map (bottom) of EWS::FLI1 CUT&Tag signal centered on tandem GGAA-AP-1 motifs annotated by Homer to EWS::FLI1 induced targets (n=578) following exposure to media (n=3), LCE (n=3), or HCE (n=3). Data demonstrates limited change in binding with either HCE or LCE. **b.** Corresponding profile plot (bottom) of average Log_2FC (treated to control) GROseq signal intensity aligned from TSS to TES to these tandem GGAA-AP-1 motifs annotated EWS::FLI1 repressed targets showing decreased nascent transcription initiation and productive transcription with LCE (n=2) but increased initiation with HCE (n=3). **c**. Identical profile plot (top) and heatmap (bottom) visualizing only *differentially bound* tandem GGAA-AP-1 motifs annotated to induced targets highlighting impaired EWS::FLI1 binding with LCE and increased occupancy with HCE. **d.** Corresponding profile plot (bottom) of average Log_2FC (treated to control) GROseq signal intensity aligned from TSS to TES of only differentially bound GGAA-AP-1 annotated EWS::FLI1 induced targets showing limited suppression of transcription initiation with LCE and HCE limited by the number of targets.

S13

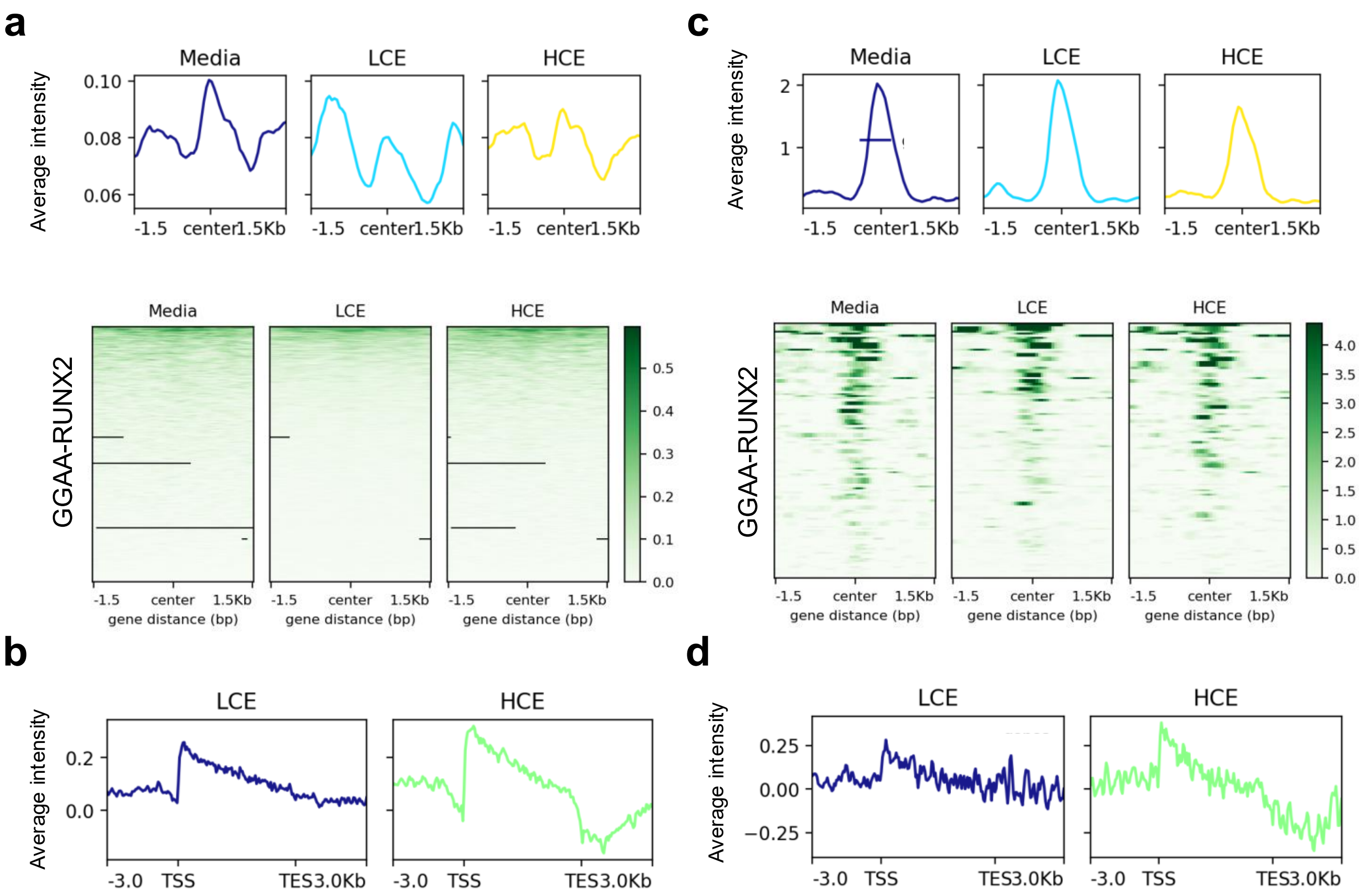

Figure S13: Loss of occupancy of EWS::FLI1 at tandem GGAA-RUNX2 sites with HCE a. Profile plot (top) of average normalized EWS::FLI1 CUT&Tag signal intensity and corresponding heat map (bottom) of EWS::FLI1 CUT&Tag signal centered on tandem GGAA-RUNX2 motifs annotated by Homer to EWS::FLI1 repressed targets (n=307) following exposure to media (n=3), LCE (n=3), or HCE (n=3). Data demonstrates small number of sites occupied by EWS::FLI1. **b.** Corresponding profile plot (bottom) of average Log_2FC (treated to control) GROseq signal intensity aligned from TSS to TES to these tandem GGAA-RUNX2 response elements annotated to EWS::FLI1 repressed targets showing increased nascent transcription initiation and productive transcription with LCE (n=2) and HCE (n=3). **c.** Profile plot (top) of average normalized EWS::FLI1 CUT&Tag signal intensity and corresponding heat map (bottom) of EWS::FLI1 CUT&Tag signal centered on *differentially bound* tandem GGAA-RUNX2 motifs annotated by Homer to EWS::FLI1 repressed targets (n=1060) following exposure to media, LCE, or HCE. Data demonstrates enrichment with LCE but loss of binding with HCE. **d.** Corresponding profile plot (bottom) of average Log_2FC (treated to control) GROseq signal intensity aligned from TSS to TES at these tandem GGAA-RUNX2 response elements annotated to EWS::FLI1 repressed targets showing minimal induction of nascent transcription initiation and productive transcription with LCE and HCE.

S14

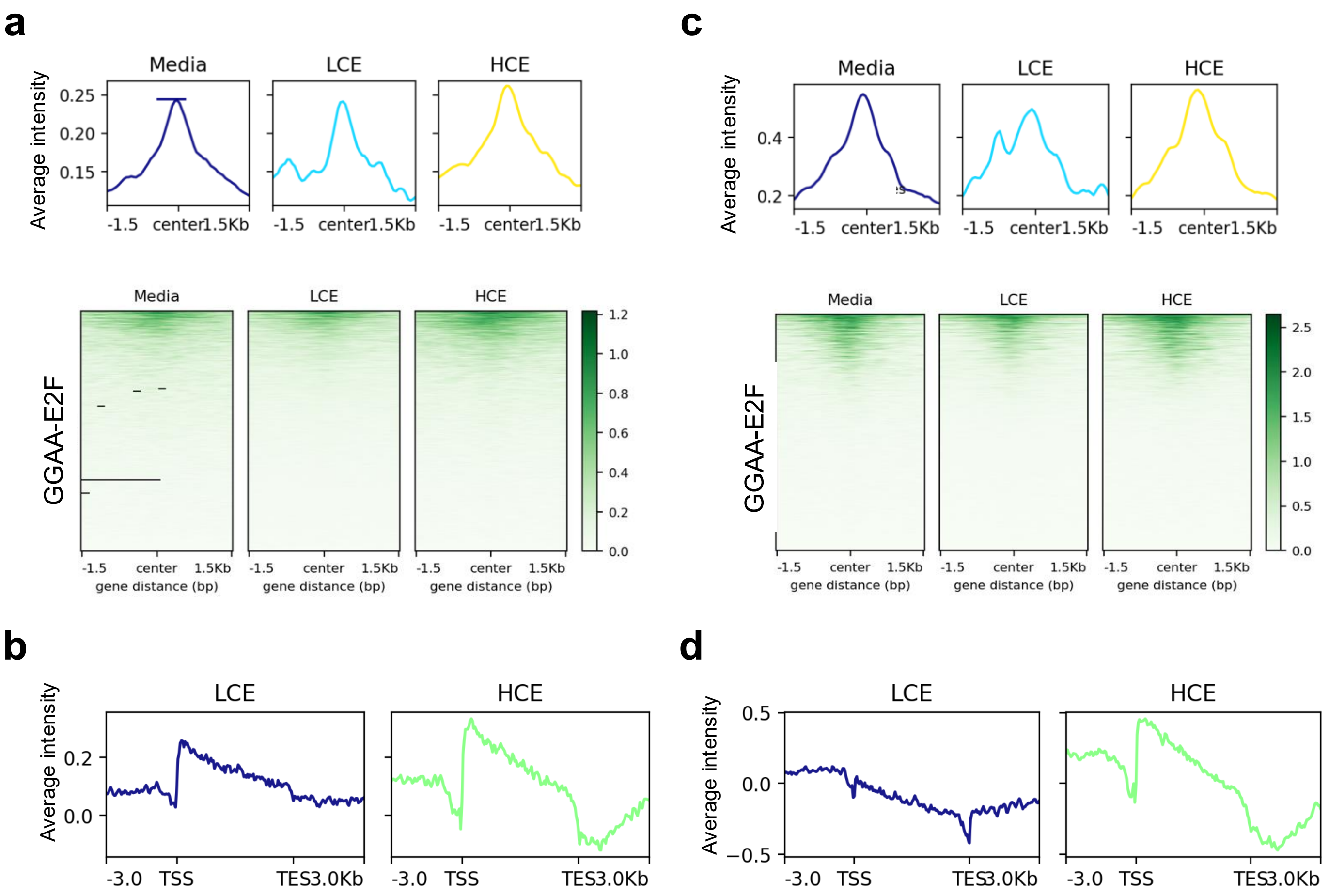

Figure S14: Loss of occupancy of EWS::FLI1 at tandem GGAA-E2F downstream target response elements with LCE and HCE mithramycin. a. Profile plot (top) of average normalized EWS::FLI1 CUT&Tag signal intensity and corresponding heat map (bottom) of EWS::FLI1 CUT&Tag signal centered on tandem GGAA-E2F motifs annotated by Homer to EWS::FLI1 repressed targets (n=487) following exposure to media (n=3), LCE (n=3), or HCE (n=3). Data demonstrates small number of sites occupied by EWS::FLI1. **b.** Corresponding profile plot (bottom) of average Log_2FC (treated to control) GROseq signal intensity aligned from TSS to TES to these tandem GGAA-E2F response elements annotated to EWS::FLI1 repressed targets showing increased nascent transcription initiation and productive transcription with LCE (n=2) and HCE (n=3). **c.** Profile plot (top) of average normalized EWS::FLI1 CUT&Tag signal intensity and corresponding heat map (bottom) of EWS::FLI1 CUT&Tag signal centered on tandem GGAA-RUNX2 motifs annotated by Homer to EWS::FLI1 induced targets (n=519) following exposure to media, LCE, or HCE. Data demonstrates loss of binding with LCE. **d.** Corresponding profile plot (bottom) of average Log_2FC (treated to control) GROseq signal intensity aligned from TSS to TES at these tandem GGAA-E2F response elements annotated to EWS::FLI1 induced targets showing loss of nascent transcription initiation and productive transcription with LCE.

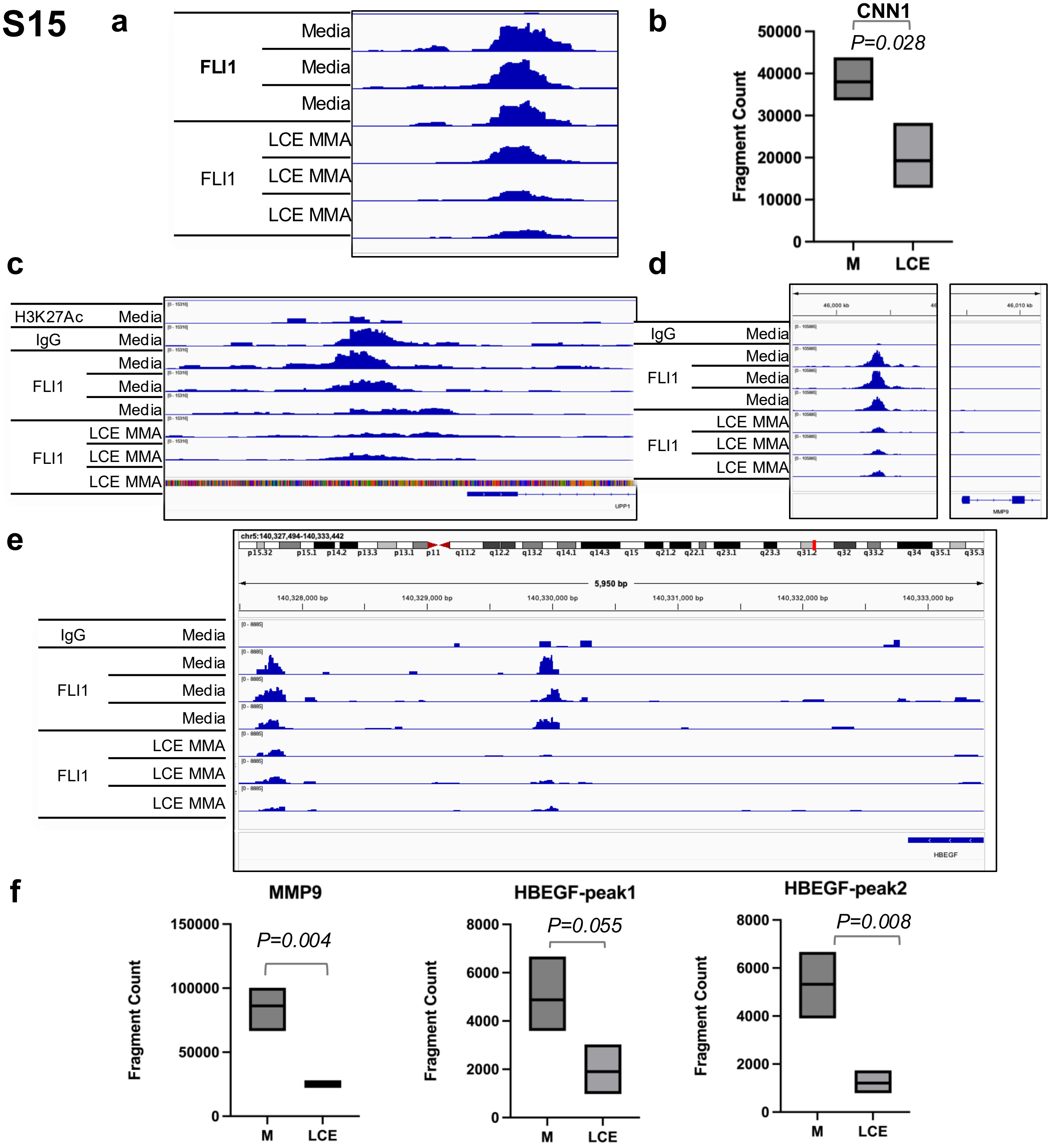

Figure S15: Mithramycin reduces binding of EWS-FLI1 containing complexes to DNA. a, IGV view of CUT&Tag data generated with a FLI1 antibody to tagment DNA following exposure to media or LCE MMA demonstrating a reduction in EWS::FLI1 binding at the tandem EWS::FLI1-RUNX locus, *CNN1*. **b**. Associated box plot showing a reduction of EWS::FLI1 binding at *CNN1* with LCE MMA (two-sided t-test, P = 0.028). **c.** IGV view of CUT&Tag data generated using the FLI1 antibody to tagment DNA following exposure to media or LCE MMA demonstrating a reduction in EWS::FLI1 binding at the tandem EWS::FLI1-AP-1 loci **c.** *UPP1* **d.** *MMP9* **e.** *HBEGF*. **f.** Associated box plot showing a reduction in EWS::FLI1 binding for the LCE exposure at *MMP9* (P = 0.004), *HBEGF* Peak 1 (P = 0.055), *HBEGF* Peak 2 (P = 0.008)(two-sided t-tests). Box plots are normalized fragment count (minimum to maximum value and the mean) comparing media (n=3) to LCE (n=3) biological replicates (see source data for full statistical analysis).

S16

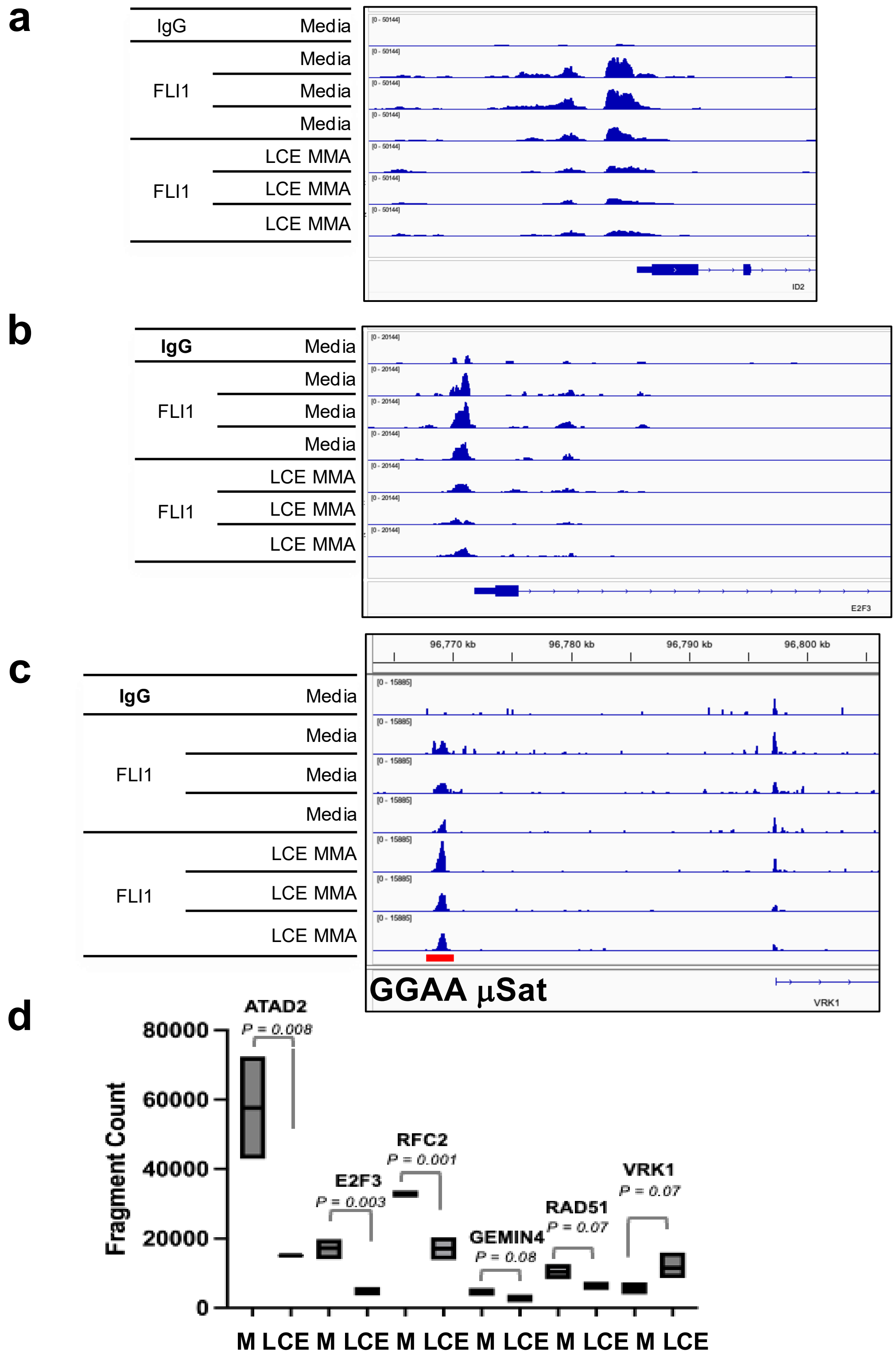

Figure S16: Mithramycin reduces binding of EWS-FLI1 at tandem E2F sites. IGV view of CUT&Tag data generated using the FLI1 antibody to tagment DNA following exposure to media or LCE MMA demonstrating a reduction in EWS::FLI1 binding at representative tandem EWS::FLI1-E2F loci **a.** *ID2* **b.** *E2F3* and **c.** *VRK1*. *VRK1* shows increased binding at a GGAA microsatellite. **d.** Box plot showing reduced EWS::FLI1 binding for media (M) vs. the LCE exposure (1-way ANOVA; $P < 0.0001$) or at all of the E2F sites: ATAD2 ($P = 0.008$), E2F3 ($P = 0.02$), RFC2 ($P = 0.003$), GEMIN4 ($P = 0.08$), RAD51 ($P = 0.07$), VRK1 ($P = 0.07$). Box plots are normalized fragment count (minimum to maximum value and the mean) comparing media (n=3) to LCE (n=3) biological replicates. (see source data for full statistics).

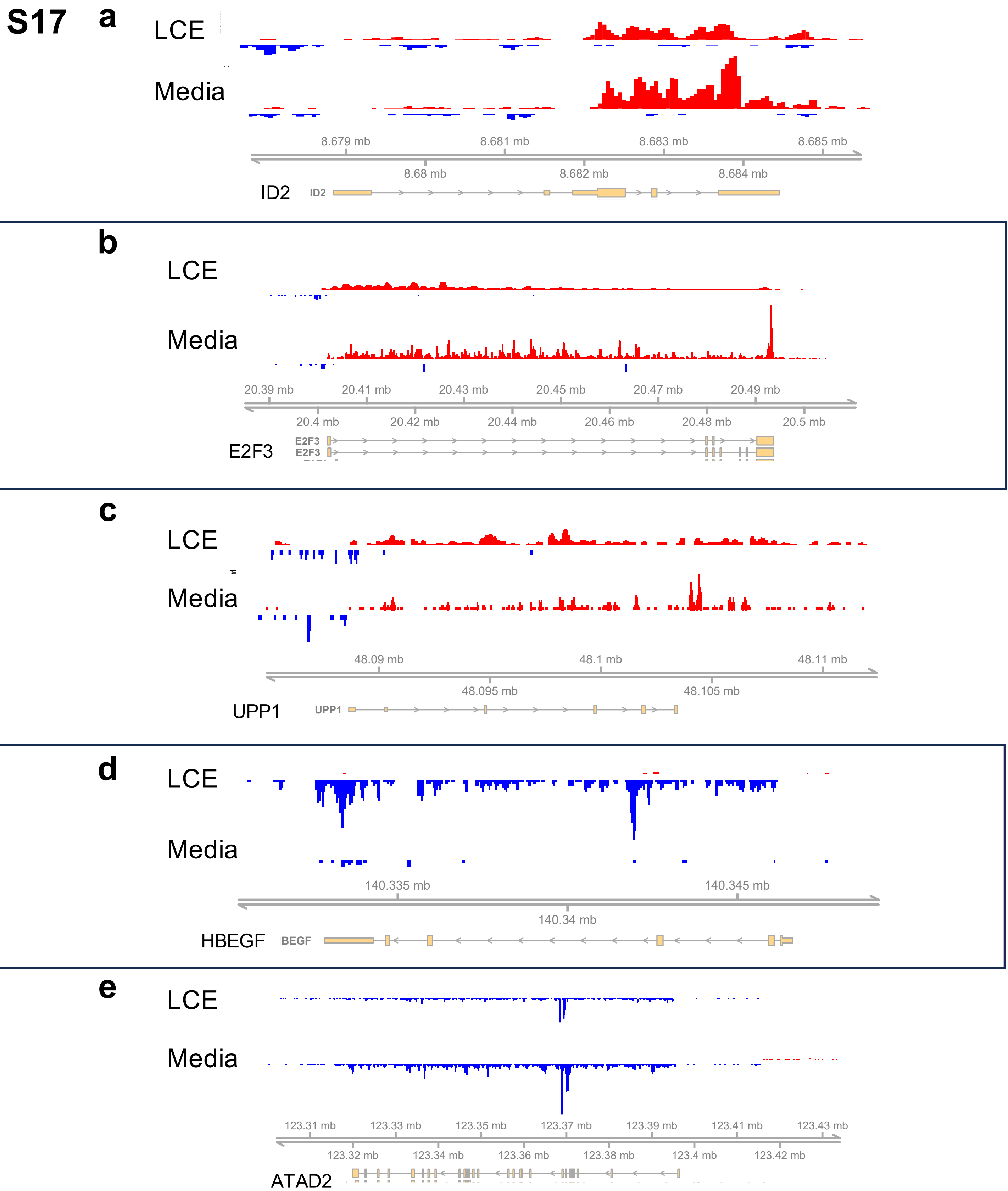

Figure S17: MMA alters nascent transcription at genes associated with loss of EWS::FLI1 binding. GROseq tracks associated with tandem EWS::FLI1 binding sites described above show altered nascent transcription following exposure to media or LCE at the **a.** *ID2* ***b.*** *E2F3* **c.** *UPP1* **d.** *HBEGF* **e.** *ATAD2* loci. Data represents the composite GROseq peak of LCE (n=2) vs. Media (n=3).

S18

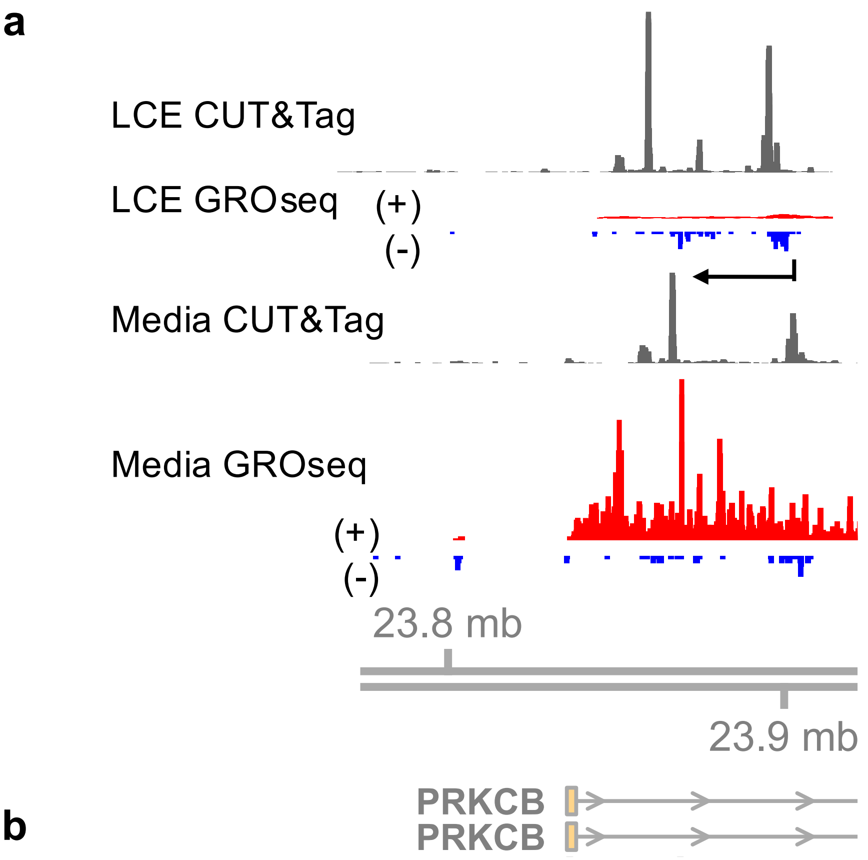

a
LCE CUT&Tag
LCE GROseq
(+)
(-)
Media CUT&Tag
Media GROseq
(+)
(-)
23.8 mb
23.9 mb
PRKCB
PRKCB

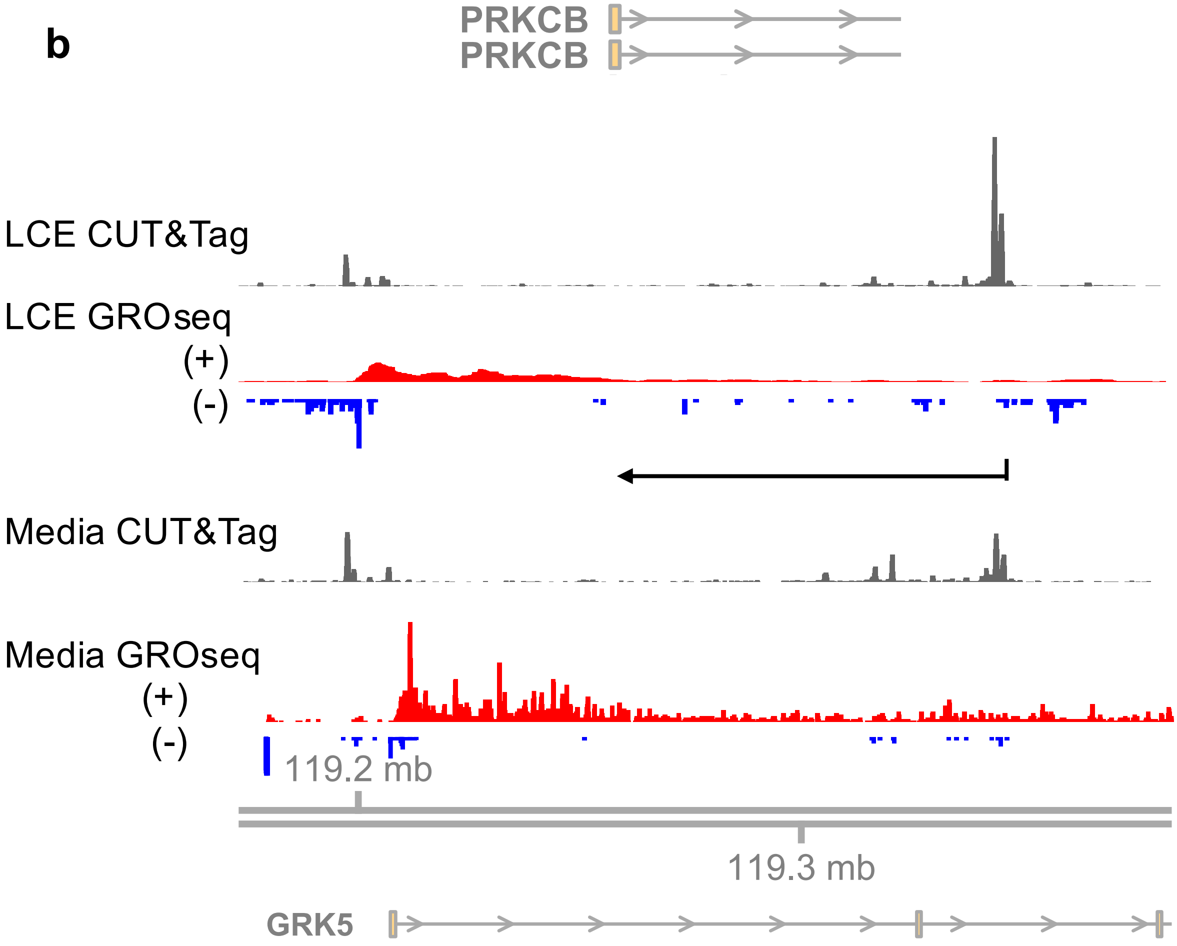

b
LCE CUT&Tag
LCE GROseq
(+)
(-)
Media CUT&Tag
Media GROseq
(+)
(-)
119.2 mb
119.3 mb
GRK5

S18 (cont'd)

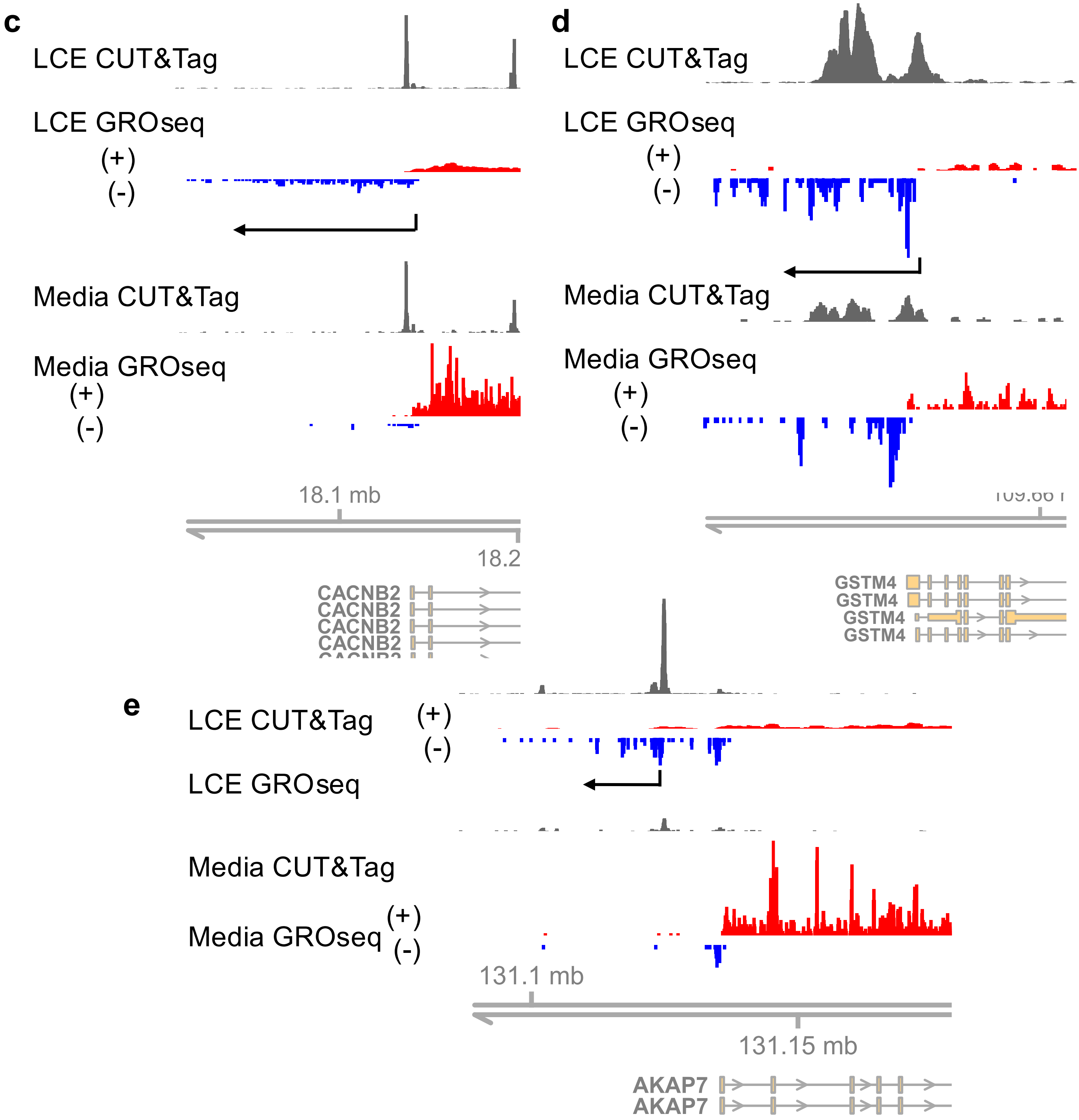

Figure S18: EWS::FLI1 initiates transcription on the opposite strand to impair transcription. Integrated CUT, Tag and GRO data demonstrating enrichment of EWS::FLI1 at downstream target genes leading to antisense transcription and possible transcription conflict with LCE MMA at **a.** *PRKCB* **b.** *GRK5* loci and initiation competition at **c.** *CACNB2* **d.** *GSTM4* and **e.** *AKAP7* loci. Arrow indicates direction of transcription initiation. All tracks have a standardized scale. Arrows indicate the direction of initiation on the opposite strand. Data represents the composite GROseq peak of LCE (n=2) vs. Media (n=3) or CUT&Tag for LCE (n=3) vs. media (n=3).

S19

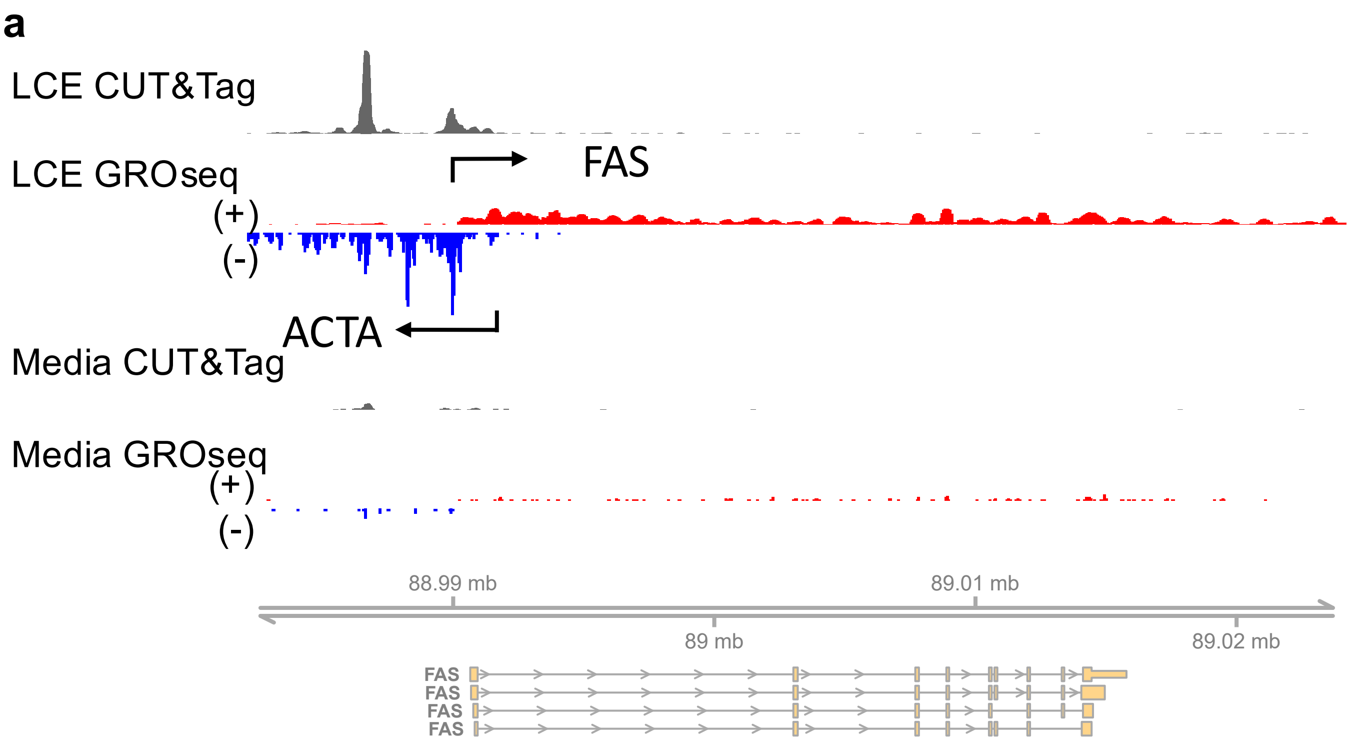
a
LCE CUT&Tag
LCE GROseq
(+)
(-)
FAS
ACTA
Media CUT&Tag
Media GROseq
(+)
(-)
88.99 mb
89.01 mb
89 mb
89.02 mb
FAS
FAS
FAS
FAS

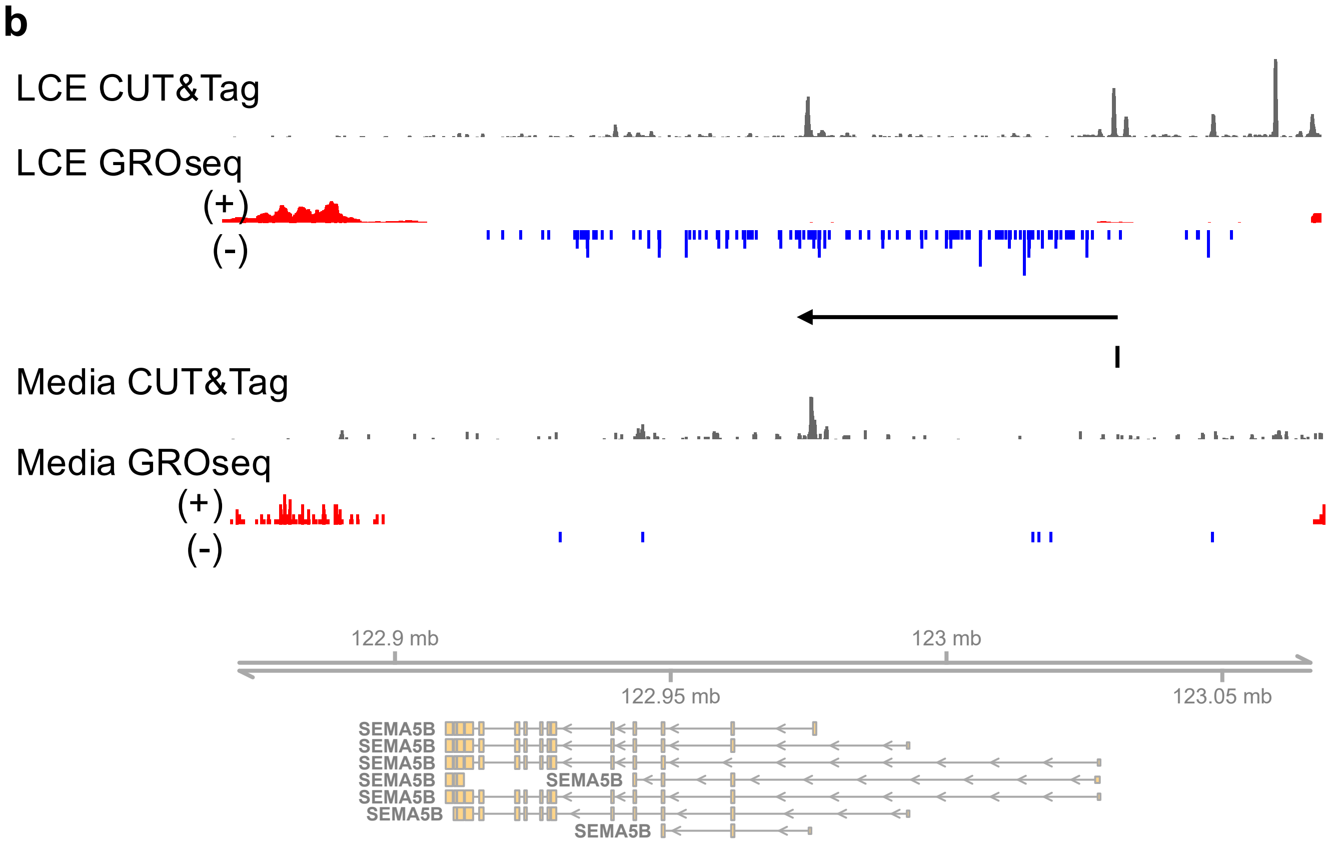
b
LCE CUT&Tag
LCE GROseq
(+)
(-)
Media CUT&Tag
Media GROseq
(+)
(-)
122.9 mb
123 mb
122.95 mb
123.05 mb
SEMA5B
SEMA5B
SEMA5B
SEMA5B
SEMA5B
SEMA5B
SEMA5B
SEMA5B
SEMA5B

S19 (cont'd)

c

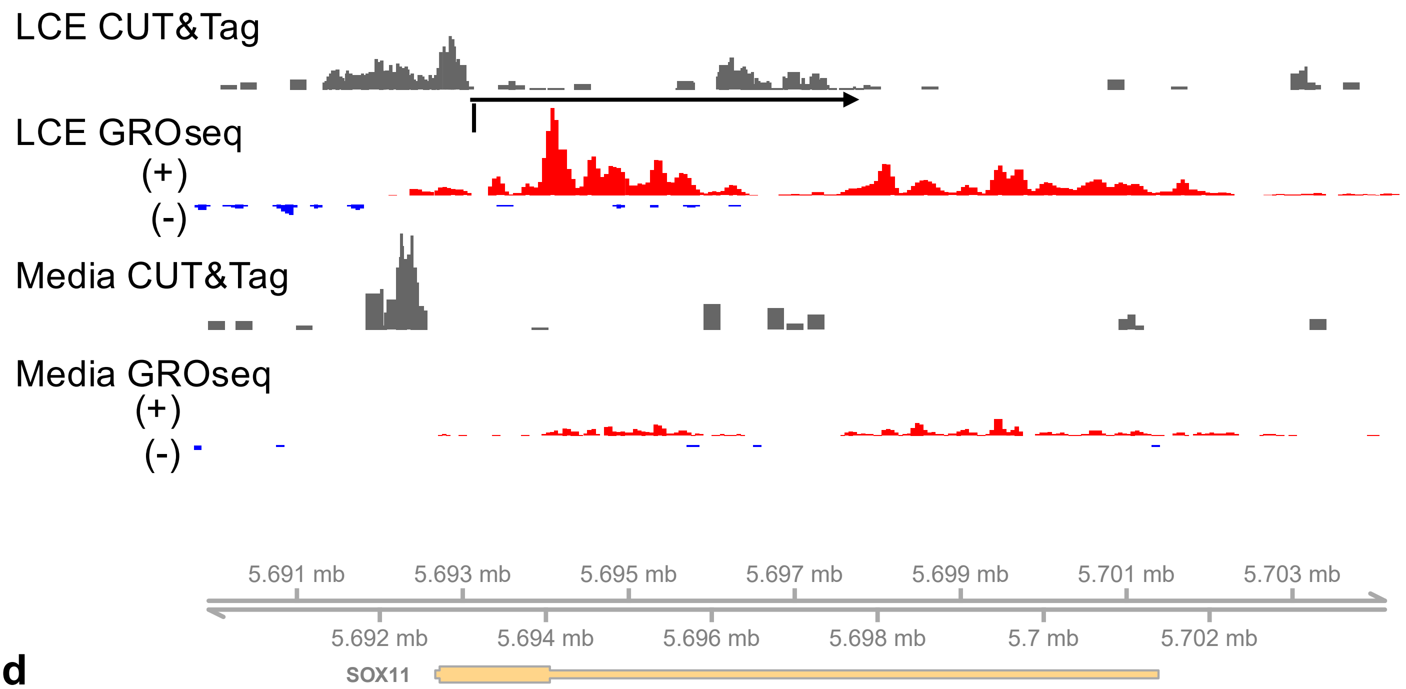

d

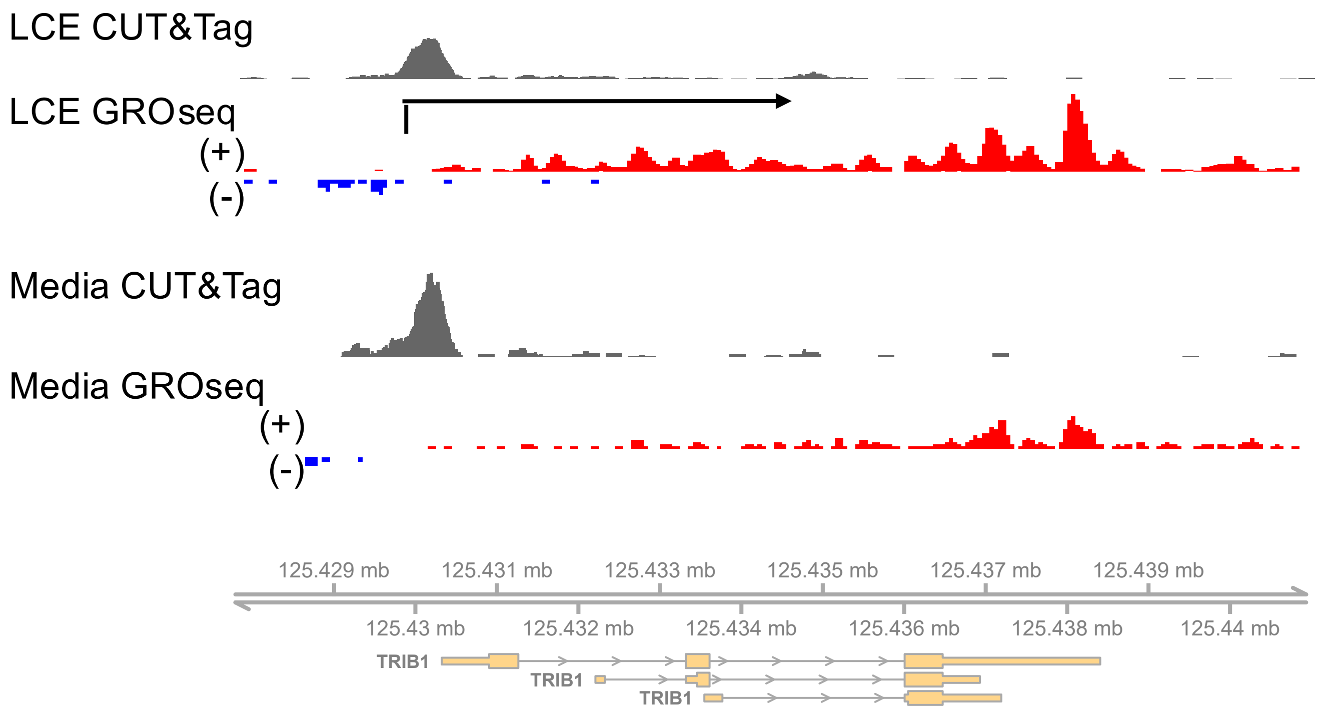

Figure S19: Enrichment or depletion of EWS::FLI1 at ETV6 competitive loci induces transcription. Integrate CUT, Tag, and GRO data demonstrating enrichment of EWS-FLI1 that overcomes the published negative regulation by ETV6 with LCE exposure to MMA at the **a.** *FAS/ACTA* **b.** *SEMA5B* and **c.** *SOX11* loci. **d.** Depletion of EWS-FLI1 at the *TRIB1* locus paradoxically induces transcription. Arrow indicates direction of transcription initiation. All tracks have a standardized scale. Data represents the composite GROseq peak of LCE (n=2) vs. Media (n=3) or CUT&Tag for LCE (n=3) vs. media (n=3).

S20

4X

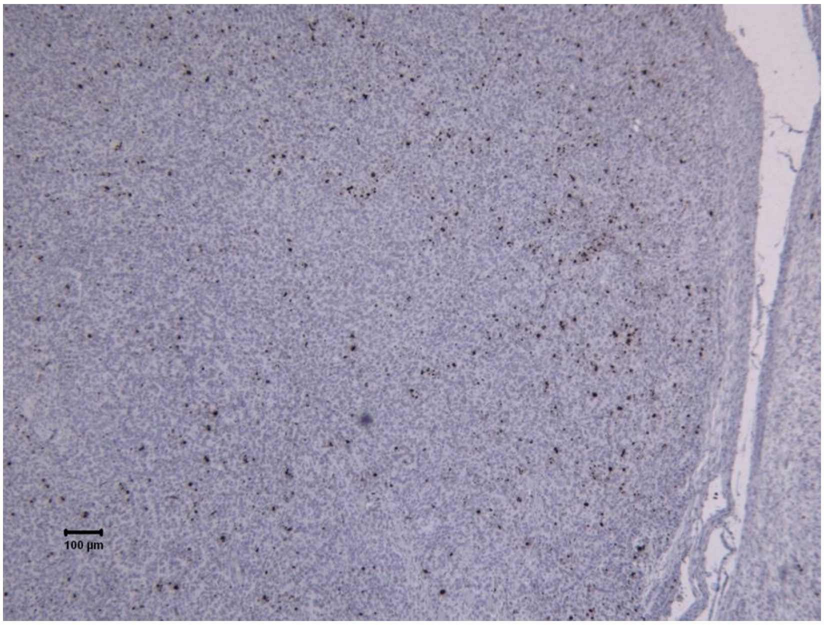

20X

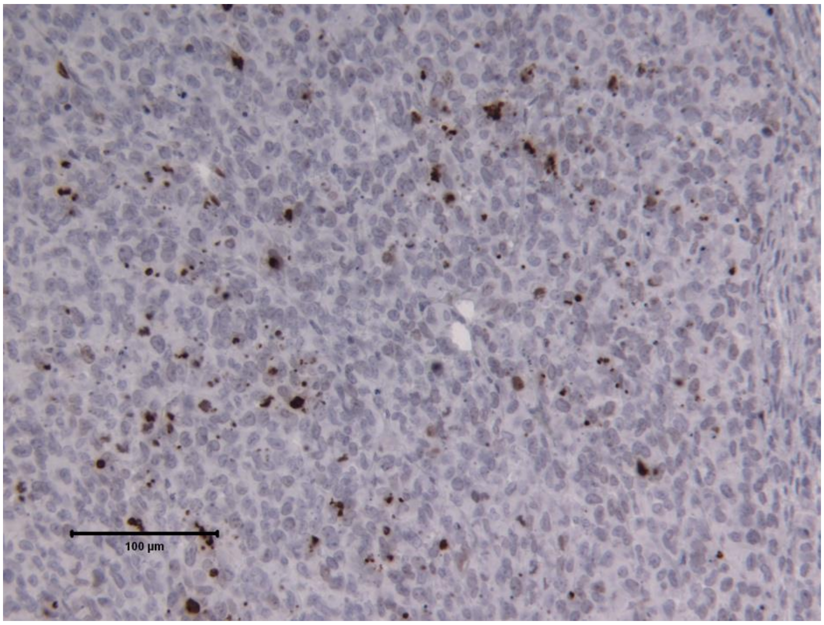

Figure S20: Mithramycin does not generate DNA damage at active doses. γH2AX staining of tissue collected on day 2 from mice treated with 0.6 mg/kg/day MMA IP X 7. There is no increase in DNA damage as measured by γH2AX staining of control tissue relative to control tissue (see Fig. 7d for associated vehicle control).

S21

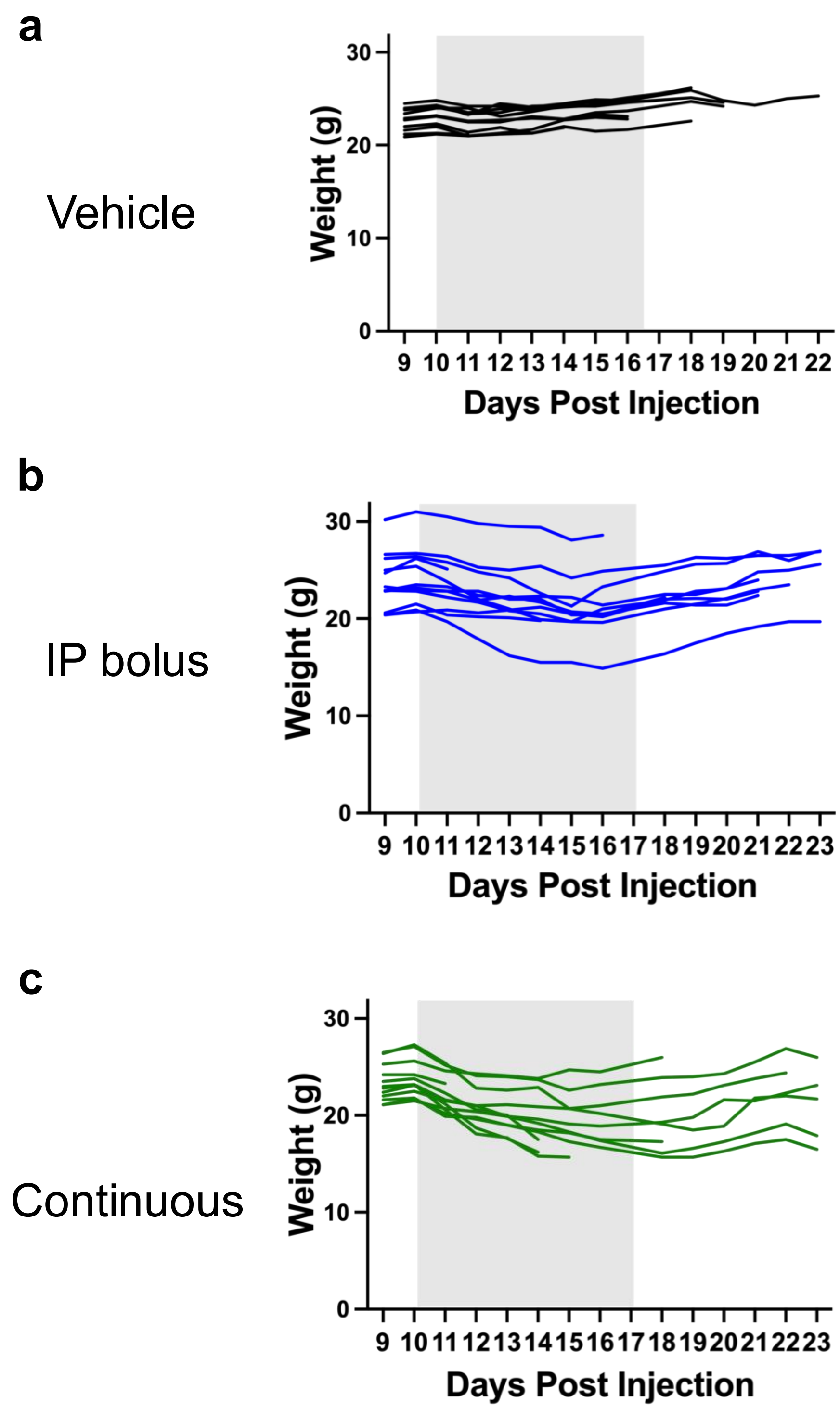

Figure S21: Minimal weight loss of mice bearing TC32 xenografts treated with mithramycin. Weights of mice bearing a TC32 xenograft treated with **a.** Vehicle (n=11) **b.** 0.6 mg/kg/day IP X 7 (n=12) or **c.** 0.6 mg/kg/day continuously (n=12). Each line represents an individual mouse; the grey box indicates duration of treatment. The difference between vehicle and either schedule is significant ($P < 0.01$) but there is no significant difference between either treatment schedule ($P = 0.65$) using the Kruskal-Wallis group comparison with Dunn correction.

S22

group	Animal ID	ALB (g/dL)	ALP (U/L)	ALT (U/L)	AMY (U/L)	TBIL (mg/dl)	BUN (mg/dL)	CA++ (mg/dL)	PHOS (mg/dL)	CRE (mg/dL)	GLU (mg/dL)	NA+ (mmol/L)	K+ (mmol/L)	TP (g/dL)	GLOB (g/dL)
v	2-00	3.3	66	25	609	0.2	18	10	5.9	0.4	147	153	5.4	5.1	1.8
v	2-01	2.9	35	31	572	0.2	20	9.7	5.4	0.5	153	151	5.4	5	2.1
v	7-00	2.5	41	39	543	0.3	~~~	9.7	6.6	<0.2*	132	148	5.6	4.7	2.3
iP	3-01	3.8	31	48	484	0.3	19	9.9	6.6	0.3	114	150	4.7	5.1	1.3
iP	6-01	3	58	105	508	<0.1	10	9.2	6.8	<0.2*	151	145	4.4	4.5	1.5
iP	6-03	3.4	36	74	564	0.3	20	10.1	5.7	0.2	179	49	5.3	5.2	1.8
7P	1-00	3	34	199	573	0.2	26	10.1	6.3	0.3	85	156	6.7	4.4	1.4
7P	1-01	3	52	230	1088	0.3	31	10.6	7.6	<0.2*	96	162	6.8	4.3	1.3
7P	4-10	2.9	40	47	698	0.2	16	9.4	7.9	<0.2*	101	148	6.8	4.8	2
7P	8-03	3.6	23	235	360	0.2	19	11	7	0.3	139	152	7.3	5.1	1.5
7P	9-01	1.8	145	288	561	0.3	21	8.3	6.6	0.3	64	151	6.5	2.7	0.9

Figure S22: **Blood chemistries indicate minimal toxicity of mice treated with MMA.** Comprehensive metabolic profile collected from animals treated with: Vehicle (group v) , 0.6 mg/kg/day IP X 7 (group iP) or 0.6 mg/kg/day continuously (group 7P) . The animals show minimal liver enzyme elevations (ALT), no sign of kidney toxicity (BUN, CRE) and minor elevations of potassium (K+) consistent with tumor lysis. Liver synthetic function is preserved as evidenced by the normal albumin and there is no evidence of hepatic insult with normal ALP and TBIL. The normal ranges are not provided by the testing agency and vary by mouse strain. Normal values across several strains are: Albumin (ALB (range 2.6-4.1)), Alkaline Phosphatase (ALP (range 41-178)), Amylase (AMY (range unknown)), Alanine transaminase (ALT (range is 38-176)), Total Bilirubin (TBIL (range 0.11 to 0.4)), Blood Urea Nitrogen (BUN (range 16-38)), Creatinine (CRE (range 0.1 to 0.4)), Glucose (GLU (range 68-348)), Total Protein (TP (range 4.4-6.1)), Globulin (Glob (range 1.7-2.6)), Sodium (Na (range145-175)), Potassium (K (range 6.5-9.7)). All reference values are from ref[55] except sodium and potassium which were in different units and so used UCLA, DLAM reference labs. Note there were no changes on histological examination of the livers.
