# Supplementary material for: Mithramycin alters EWS::FLI1 DNA binding and RNA polymerase II processivity to inhibit nascent transcription
Source: Nat Commun. 2026 Feb 16;17:2844. doi: 10.1038/s41467-026-69488-9 (PMC13021929; doi:10.1038/s41467-026-69488-9)
Supplement: Supplementary file 2 — Description of Additional Supplementary Files [file 41467_2026_69488_MOESM2_ESM.pdf]

Description of Additional Supplementary Files

Supplementary Data 1. Reagents and RRID Table and Primers
