# Supplementary material for: Mithramycin alters EWS::FLI1 DNA binding and RNA polymerase II processivity to inhibit nascent transcription
Source: Nat Commun. 2026 Feb 16;17:2844. doi: 10.1038/s41467-026-69488-9 (PMC13021929; doi:10.1038/s41467-026-69488-9)
Supplement: Supplementary file 4 — Reporting Summary [file 41467_2026_69488_MOESM4_ESM.pdf]

nature portfolio

Corresponding author(s): Patrick J. Grohar

Last updated by author(s): Jan 23, 2026

Reporting Summary

Nature Portfolio wishes to improve the reproducibility of the work that we publish. This form provides structure for consistency and transparency in reporting. For further information on Nature Portfolio policies, see our Editorial Policies and the Editorial Policy Checklist.

Please do not complete any field with "not applicable" or n/a. Refer to the help text for what text to use if an item is not relevant to your study.
For final submission: please carefully check your responses for accuracy; you will not be able to make changes later.

Statistics

For all statistical analyses, confirm that the following items are present in the figure legend, table legend, main text, or Methods section.

n/a	Confirmed	
☐	☒	The exact sample size (*n*) for each experimental group/condition, given as a discrete number and unit of measurement
☐	☒	A statement on whether measurements were taken from distinct samples or whether the same sample was measured repeatedly
☐	☒	The statistical test(s) used AND whether they are one- or two-sided<br>*Only common tests should be described solely by name; describe more complex techniques in the Methods section.*
☒	☐	A description of all covariates tested
☐	☒	A description of any assumptions or corrections, such as tests of normality and adjustment for multiple comparisons
☐	☒	A full description of the statistical parameters including central tendency (e.g. means) or other basic estimates (e.g. regression coefficient) AND variation (e.g. standard deviation) or associated estimates of uncertainty (e.g. confidence intervals)
☐	☒	For null hypothesis testing, the test statistic (e.g. *F*, *t*, *r*) with confidence intervals, effect sizes, degrees of freedom and *P* value noted<br>*Give P values as exact values whenever suitable.*
☒	☐	For Bayesian analysis, information on the choice of priors and Markov chain Monte Carlo settings
☒	☐	For hierarchical and complex designs, identification of the appropriate level for tests and full reporting of outcomes
☒	☐	Estimates of effect sizes (e.g. Cohen's *d*, Pearson's *r*), indicating how they were calculated

Our web collection on statistics for biologists contains articles on many of the points above.

Software and code

Policy information about availability of computer code

Data collection	Custom code was not used for data collection. Full description of analysis is listed below.
Data analysis	Genomics Data Analysis: GROseq: Following adapter trimming and low quality sequence removal by cutadapt v2.1064, GROseq reads were aligned to hg38 using STAR v2.7.1a65. Sequence quality was additionally confirmed by fastqc. Additional quality control for GROseq was done using NRSA tools66. Reads mapping to rRNA loci and reads with mapping quality less than 60 were removed. After read mapping and filtration, reads around the TSS (+30bp,-300bp),TES (TES-30bp, +300bp) and Gene Body (TSS+30bp,TES-30bp) of genes were quantified using bedtools coverage v2.29.267. Heatmaps were generated with deeptools v3.5.168. For visualization, log2ratio GRO-seq signal of treatment vs. media (or solvent) was calculated using deeptools bamCompare. All tracks were group scaled to the highest peak in control.<br>CUT&Tag bioinformatics and motif analysis: Sequence quality was first assessed with FastQC (v0.12.1). Sequencing reads were trimmed to remove adapter sequences and low-quality bases using Trim Galore (v0.6.6). Trimmed reads were then aligned to hg38 with bowtie2 v2.3.4.2 with parameters "--local --very-sensitive --no-mixed --no-discordant --phred33 -I 10 -X 700" Aligned read pairs on the same chromosome and fragment length less than 1000bp were kept and filtered on minQualityScore=2 with samtools v1.15. Peaks were called with macs2 v2.2.4 callpeak and peaks in blacklist regions were removed with bedtools intersect. Only uniquely mapping reads were retained. Reproducibility between replicates was assessed by computing pairwise Spearman correlations of signal intensities using R corrplot. Peaks were called using MACS2 with a q-value threshold of 0.05, then Fraction of Reads in Peaks (FRiP) was calculated using bedtools intersect to quantify signal enrichment. DiffBind v 3.16.0 was used to identify differentially bound peaks (padj < 0.05) in LCE,HCE,and LCE vs HCE. (HCE vs Media n=10475, LCE vs Media n=35119, HCE vs LCE n= 15952). Differentially bound peaks were then further characterized for their associated motifs using Homer for both LCE and HCE. Identification of EWS::FLI1 induced or repressed targets by RNAseq: EWS::FLI1 was silenced using an siRNA spanning the fusion breakpoint in biological replicates of 3 per condition using a previously published approach40 and confirmed by qPCR and a 3-cycle (23) change in expression. siEWS::FLI RNA-seq reads were mapped to hg38 with STAR v2.7.1a65 and transcript expression was quantified with RSEM v1.3.169. Following quantification, induced and repressed targets were identified with DESeq2 v1.32.070. using a log

fold change cutoff of +/- 1 and adjusted P-value cutoff of 0.01. These lists of induced and repressed targets were used to annotate CUT&Tag peaks below.
EWS::FLI1 differential binding analysis: Tandem motif identification: Genome wide motif locations for AP-1 (n = 78183) RUNX2 (n = 72469), E2F3 (n = 82248), and FLI1 (n = 73629) were extracted using fimo v 5.5.8. Isolated GGAAs not within 20bp of another GGAA were found using BioStrings (n = 18377396). Isolated GGAAs intersecting with a differentially bound peak were retained (n = 137254). The resulting GGAAs were filtered within 200bp of cofactor motifs AP-1 (n = 7737), RUNX2 (n = 2207), E2F3 (n = 4130) or FLI1 (n = 9855). Peak annotation of tandem motifs or FLI1 to EWS::FLI1 induced or repressed targets: Homer annotatePeaks.pl was used to annotate motifs and peaks and were filtered to include GGAAs for EWS::FLI1 induced targets (Log2FC < 1; P < 0.01)(GGAA-AP-1 n=578, GGAA-RUNX2 n = 197; GGAA-E2F3 n = 519, FLI1 n = 857) or EWS::FLI1 repressed targets (Log2FC >1; P <0.01)(GGAA-AP-1 n = 1106 , GGAA-RUNX2 n = 307 ; GGAA-E2F3 n = 487 , FLI1 n = 1060).
Microsatellite identification and annotation to EWS::FLI1 induced or repressed targets: GGAA microsatellites were identified using fimo v 5.5.8 (n = 20,765), annotated to genes using Homer annotatePeaks.pl, then filtered on EWS::FLI1 induced (GGAA μSat n = 445) and EWS::FLI1 repressed (GGAA μSat n = 487) targets. Visualization of EWS::FLI1 binding: CUT&Tag signal at these motifs and GRO-seq Log2ratio signal at annotated genes were visualized with deeptools computeMatrix, plotHeatmap and plotProfile (v 3.5.6). For the diff_bind graphs only differentially bound peaks were visualized. All statistical analyses were performed using GraphPad Prism (v 10.6.1).

For manuscripts utilizing custom algorithms or software that are central to the research but not yet described in published literature, software must be made available to editors and reviewers. We strongly encourage code deposition in a community repository (e.g. GitHub). See the Nature Portfolio guidelines for submitting code & software for further information.

Data

Policy information about availability of data

All manuscripts must include a data availability statement. This statement should provide the following information, where applicable:

- Accession codes, unique identifiers, or web links for publicly available datasets
- A description of any restrictions on data availability
- For clinical datasets or third party data, please ensure that the statement adheres to our policy

All genomic data will be deposited in GEO prior to publication

Research involving human participants, their data, or biological material

Policy information about studies with human participants or human data. See also policy information about sex, gender (identity/presentation), and sexual orientation and race, ethnicity and racism.

Reporting on sex and gender	The reported research does not involve human participants or their data
Reporting on race, ethnicity, or other socially relevant groupings	The reported research does not involve human participants or their data
Population characteristics	The reported research does not involve human participants or their data
Recruitment	The reported research does not involve human participants or their data
Ethics oversight	The reported research does not involve human participants or their data

Note that full information on the approval of the study protocol must also be provided in the manuscript.

Field-specific reporting

Please select the one below that is the best fit for your research. If you are not sure, read the appropriate sections before making your selection.

☒ Life sciences ☐ Behavioural & social sciences ☐ Ecological, evolutionary & environmental sciences

For a reference copy of the document with all sections, see nature.com/documents/nr-reporting-summary-flat.pdf

Life sciences study design

All studies must disclose on these points even when the disclosure is negative.

Sample size	Sample size was chosen based on previous work to achieve >80% power between two treatment groups with additional animals included for tissue collection and analysis
Data exclusions	All data was included in the manuscript. One replicate for the GROseq was excluded due to sample failure.
Replication	All data is repeated in a minimum of three independent experiments, in most cases by two different individuals in the lab.
Randomization	Randomization was performed for all in vivo experiments after tumor establish but prior to treatment and matched for tumor size across cohorts.

Blinding	Blinding was not possible, but groups were randomized.

Reporting for specific materials, systems and methods

We require information from authors about some types of materials, experimental systems and methods used in many studies. Here, indicate whether each material, system or method listed is relevant to your study. If you are not sure if a list item applies to your research, read the appropriate section before selecting a response.

Materials & experimental systems

n/a	Involved in the study
☐	☒ Antibodies
☐	☒ Eukaryotic cell lines
☒	☐ Palaeontology and archaeology
☐	☒ Animals and other organisms
☒	☐ Clinical data
☒	☐ Dual use research of concern
☒	☐ Plants

Methods

n/a	Involved in the study
☒	☐ ChIP-seq
☒	☐ Flow cytometry
☒	☐ MRI-based neuroimaging

Antibodies

Antibodies used	GAPDH Abcam ab8245<br>FLI1 Abcam ab133485<br>NR0B1 Abcam ab196649<br>RPB1 Cell Signaling 2629<br>EZH2 Cell Signaling 5246<br>DPF2 Cell Signaling 71642<br>H3 Cell Signaling 3638<br>PBRM1 Cell Signaling 89123<br>BRD9 Cell Signaling 71232<br>anti-mouse Cell Signaling 7076<br>anti-rabbit Cell Signaling 7074<br>gamma H2aX Cell Signaling 9718<br>Goat Anti-Rabbit Immunoglobulins/HRP Agilent P0448<br>BrdU Antibody (IIB5), Agarose Conjugated Beads Santa Cruz sc-32323- ac<br>Histone H3K27ac Active Motif 39135<br>Normal Rabbit IgG Cell Signaling 2729<br>Anti-FLI1 Abcam ab15289
Validation	Antibodies have been validated using positive and negative controls and compared to samples with known expression levels. Additional validation is performed using antibody titration. IHC tissues have optimized antigen retrieval HIER and have been validated with the use of positive and negative control tissues.

Eukaryotic cell lines

Policy information about cell lines and Sex and Gender in Research

Cell line source(s)	TC32 cells were obtained from Dr. Lee Helman (Osteosarcoma Institute), TC252 cells were a gift from Dr. Tim Triche (Children's Hospital of Los Angeles, Los Angeles, CA).
Authentication	Cell lines were authenticated by short tandem repeat profiling (DDC Medical and Penn Genomic and Sequencing Core).
Mycoplasma contamination	Cell lines were routinely screened for mycoplasma (Penn Genomic Sequencing Core)
Commonly misidentified lines<br>(See ICLAC register)	none

Animals and other research organisms

Policy information about studies involving animals; ARRIVE guidelines recommended for reporting animal research, and Sex and Gender in Research

Laboratory animals	Athymic nude mice Crl; Nu-Foxn1Nu
Wild animals	No wild animals were used in the reported studies.

Reporting on sex	Findings are reported for female mice.
Field-collected samples	Our studies did not include field-collected samples.
Ethics oversight	All experiments were performed in accordance with and the approval of the Van Andel Institute Institutional Animal Care and Use Committee or University of Michigan's Unit for Laboratory Animal Medicine IACUC.

Note that full information on the approval of the study protocol must also be provided in the manuscript.

Plants

Seed stocks	Plants were not used in the reported research.
Novel plant genotypes	Plants were not used in the reported research.
Authentication	Plants were not used in the reported research.

This checklist template is licensed under a Creative Commons Attribution 4.0 International License, which permits use, sharing, adaptation, distribution and reproduction in any medium or format, as long as you give appropriate credit to the original author(s) and the source, provide a link to the Creative Commons license, and indicate if changes were made. The images or other third party material in this article are included in the article's Creative Commons license, unless indicated otherwise in a credit line to the material. If material is not included in the article's Creative Commons license and your intended use is not permitted by statutory regulation or exceeds the permitted use, you will need to obtain permission directly from the copyright holder. To view a copy of this license, visit http://creativecommons.org/licenses/by/4.0/
